# The functional foetal brain: A systematic review and meta-analysis

**DOI:** 10.1016/j.dcn.2026.101708

**Published:** 2026-03-17

**Authors:** Leonie Loehn, Kirsty Dunn, Michelle To, Vincent M. Reid

**Affiliations:** a The University of Waikato, School of Psychology and Social Sciences, Private Bag 3105, Hamilton 3240, New Zealand; b Lancaster University, Department of Psychology, Tower Avenue, Bailrigg, Lancaster LA1 4XX, United Kingdom

**Keywords:** Foetus, Visual perception, Auditory perception, Functional magnetic resonance imaging (fMRI), Foetal magnetoencephalography (fMEG), Evoked responses

## Abstract

This work examines functional magnetic resonance imaging and foetal magnetoencephalography studies that measure foetal auditory and visual evoked responses during the third trimester of gestation. A prior study (Dunn et al., 2015) found inconsistent results in healthy human samples due to substantial methodological variability. The updated review also includes studies where participants are at risk of, or are already showing, atypical development. This gives a broader understanding of how and when sensory abilities develop and in which way their development is influenced by foetal and maternal risk factors. 46 studies were included from initial 528 reports. They demonstrate that auditory stimuli activate primarily temporal areas whereas visual stimuli activate only frontal regions. They also show that latencies and amplitudes of foetal evoked responses are comparable between both modalities, but are negatively affected by atypical development. Several stimulus and paradigm characteristics further moderate latency and amplitude values modality specific. Due to insufficient data regarding the gestational age, it remains inconclusive how age affects latencies and amplitudes. Older foetuses show, however, increased response rates and decreased latencies compared to younger foetuses, independent of modality. Key recommendations relate to improving data quality and comparability between studies. This will enable the development of clinical assessment tools in the future.

## Highlights

• Auditory stimuli primarily activate areas in the temporal lobes.
• Visual stimuli activate only frontal brain regions.
• Latencies and amplitudes of evoked responses are comparable between modalities.
• Stimulus and paradigm characteristics moderate foetal evoked responses.
• Older foetuses have higher response rates and lower latencies than younger foetuses.

## 1 Introduction

How does the human foetus perceive the world? How does the foetus respond to external sensory stimuli? And how do we know whether a foetus is responding in a typical manner, thereby indicating healthy development?

In 1985, Blum et al. laid the foundations for addressing these questions by performing the first direct recording of foetal brain responses to externally presented sound. Neural correlates of auditory stimulation were measured using foetal magnetoencephalography (fMEG). Up until then, only indirect measures of foetal brain activity were available, such as cardiotocography to measure the foetal heartrate or ultrasound to observe foetal breathing and movement patterns (e. g. Schmidt et al., 1985). Several studies have followed since using either fMEG or functional magnetic resonance imaging (fMRI) to measure foetal brain activity after sensory stimulation.

Owing to the difficulty in refining fMEG and the expense of this technology, there has only been slow progress in the field over the past 30 years. In 2015, Dunn et al. summarised fMRI and fMEG studies that measured foetal evoked responses (fER) after auditory and visual stimulation in typically developing foetuses. The main purpose of the review was to explore the coherence of methodological practices across studies and to determine how these factors contributed to the heterogeneity in the field. The authors covered 26 articles with 29 studies and compared auditory and visual studies separately. They showed that it is possible to measure fER after visual and auditory stimulation with both fMRI and fMEG methodologies using a wide range of study designs. Further, methodological variability between studies influenced the response rate and the latency of fER. There was also a large variability in the comprehensiveness of how researchers reported their methodologies and results, effectively limiting comparisons between different approaches. The authors noted that the field remained in its infancy and that many studies have focused on the feasibility of their designs or analysis techniques rather than utilizing standardized protocols. Dunn et al. (2015) consequently recommended the establishment of a standardized protocol and advised researchers to consistently report as much of their procedure, data handling, and results as possible. The authors concluded that these recommendations should not only improve the likelihood of individual studies to successfully measure foetal brain responses, but they should also facilitate comparisons between different approaches. This would eventually enable a better understanding of how foetuses perceive the world by better understanding how they respond to auditory and visual stimulation.

The present review provides an update to Dunn et al. (2015) to explore how the field has developed over the last decade. Of particular interest was whether the above-listed recommendations had been implemented in those studies published over the last ten years. Understanding the emergence and nature of foetal sensory abilities is important as it is well known that prenatal experiences shape later development (e. g. Pallas, 2005; Panneton et al., 2021). To date, the field has only limited knowledge about foetal sensory development. Variation in results from existing studies further limits our understanding of early human development. The present review aims to address these issues by synthesising the entire field of foetal sensory fMEG and fMRI studies. Such an approach seeks to understand these published studies in context to each other, rather than looking at them separately. The results from this synthesis provide a platform for drawing more generalised conclusions about which (sensory) abilities are present in the human foetus. Using a meta-analytical approach, this up-to-date review looks to determine which methodological factors contribute to heterogenous results between studies. Through so doing, this article may enable us to understand which sample, stimulus, and paradigm characteristics make foetal responses to environmental stimuli more likely to be measured by cognitive neuroscience techniques. The current work goes beyond Dunn et al. (2015) by using a meta-analytical approach to quantitatively investigate the influence of these methodological factors on latency and amplitude values of fER when data was sufficient to do so. The review was also expanded to determine how atypical development moderates foetal brain responses to sensory stimulation by including samples with either maternal or foetal risk factors, such as gestational diabetes or intrauterine growth restriction. In addition, each study was rated on twelve dimensions of data quality which covered the research objective, the sample, the study design/ methodology, the results, and possible biases. Thus, the present review seeks to provide an up-to-date overview of the field of fMRI and fMEG studies related to foetal sensory stimulation which will help the field to gain a greater understanding of early human ontogenetic development.

## 2 Methods

The approach that was taken to identify, screen, and finally select suitable studies, as well as the pathways that guided data extraction, followed PRISMA guidelines (Page et al., 2021) and were based on the processes implemented by Dunn et al. (2015).

### 2.1 Identification of eligible studies

On the 19th of October 2023, five databases were searched for the identification of eligible studies with either the search string “fetal AND (fMRI OR fMEG)” (APA PsychNet, ProQuest Social Sciences, & Scopus) or the search string “fetal AND (fMRI OR fMEG) AND evoked response” (PubMed & Science Direct). The longer search string was chosen to decrease the large number of irrelevant results in the latter two databases. 528 records were identified through this in total.

After duplicates were removed and only journal articles remained, 399 results were screened for eligibility based on the following inclusion criteria: Investigation of the human foetus, using fMRI/ fMEG methodologies, auditory/ visual/ olfactory/ gustatory/ touch perception, singleton pregnancies, original dataset, reported since 1985, and published in a peer-reviewed English language journal.

The corresponding exclusion criteria were: Non-foetal sample, non-human sample, no fMRI/ fMEG methodology, investigation of only resting state/ steady state responses, multifoetal pregnancy, genetic study (studies investigating gene functions or gene expression), method paper (articles focusing on the methodology of fMRI, fMEG or other neuroimaging methods without reporting foetal brain responses), non-original dataset, not written in English, summary of abstracts, review.

In total, 65 records matched the above criteria during abstract screening. These were then further screened upon examination of the full text with the following additional exclusion criteria: No full text available, no evoked responses investigated, solely comparison of data analysis techniques, data reported incompletely, and unreliable methodology due to methodological confounds.

**Fig. 1:**
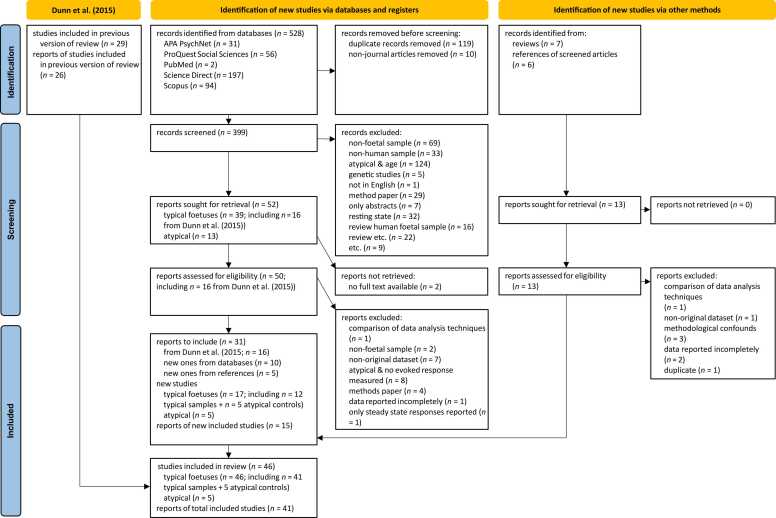
PRISMA Flow-chart. *Note*. The number of studies in Dunn et al. (2015) refers to the number of studies based on the present criteria. Muenssinger et al. (2013a) was split into two studies here as they described two separate studies in their article. Dunn et al. (2015) had counted the two studies as one. The earliest three feasibility studies (Blum et al., 1985, Wakai et al., 1996, Eswaran et al., 2000) were excluded from the analyses in Dunn et al. (2015) due to possible methodological confounds, e. g. environmental noise (Lengle et al., 2001). These three studies were therefore not counted as included in Dunn et al. (2015) since they were not part of the author’s summaries.

Based on these criteria, 41 articles containing 46 studies were included in the final dataset (see flow chart below, Fig. 1). Of those, 29 were already summarised in the preview by Dunn et al. (2015). 17 new studies were identified, including five studies that investigated samples with maternal or foetal risk factors, such as gestational diabetes or intrauterine growth-restriction. There were ten studies examining foetal visual responses, one using fMRI and nine using fMEG. Foetal auditory responses were investigated more extensively with a total of 36 studies that were included in the present review. Six of those were fMRI studies and 30 used fMEG methodologies.

### 2.2 Extraction of data

Table 1 shows the factors of extracted data from the included studies.

**Table 1:** Extracted data of included studies.

sample characteristics	stimulus characteristics	paradigm characteristics	outcome variables
− number of recruited participants − number of analysed participants − attrition rate − gestational age (range, M, & SD)	− stimulus type − stimulus duration − interstimulus interval (ISI) − ratio of stimulus duration/ ISI − frequency (auditory stimuli)/ wavelength (visual stimuli) − stimulus intensity	− paradigm − delivery method − number of presentations − mean experimental time	− number of trials included in analyses − response rate − age effect − activated brain area (fMRI studies) − response latency (fMEG studies) − response amplitude (fMEG studies) − early & late responses (fMEG studies)

**Note:***M* = mean, *SD* = standard deviation, fMRI = functional magnetic resonance imaging, fMEG = foetal magnetoencephalography. The attrition rate is the percentage of the analysed sample divided by the number of recruited participants.

The main outcome variables in fMRI studies were the activated brain areas and the response rate, which refers to the percentage of analysed participants for which an evoked response was measured. In fMEG studies, the main outcome variables were the response rate, the response latency, and the response amplitude. The response latency describes the time in milliseconds from when a stimulus is presented until an evoked response can be measured in the foetal brain. The response amplitude shows the size of the evoked response in femtotesla (fT). It indicates the maximum shift in brain activity caused by the (initial) processing of the presented stimulus.

### 2.3 Dealing with missing data

Missing values were calculated whenever possible. If the information provided was insufficient to do so, then the corresponding author was contacted at least once to provide more details. If authors did not respond, their study was either excluded from the review (*n* = 11) or excluded from certain analyses as a function of how much, and which, information was missing or incomplete (*n* = 7).

### 2.4 Quality assessment

The studies were rated for their quality using twelve questions (see quality assessment form, appendix A in the supplementary material), which were based on the Specialist Unit for Review Evidence (SURE) checklist for cross-sectional studies (2018). The quality assessment covered the research question, the participant selection, the study sample, the study design (stimulus and paradigm characteristics), the methodology, the estimation of sample size, the analysed sample size, the results (completeness, description, raw data availability), and conflicts of interest.

Each individual question was rated from “very good” (all information provided; score: 6) to “missing”/ “not stated” (no information provided; score: 0). The responses were then summarised to calculate an average score for the quality of a study. A rating could also be adjusted, depending on how the individual questions were answered. For example, it could be lowered when the results were based on a very low sample size, e. g. *N* = 3 (Jardri et al., 2008) as a low sample size typically leads to less informative results than a study with many participants, where reported results would likely be more robust. The rating of the research question was not considered for the average quality rating as all research questions were rated based on the topic of the present review while the investigation of fER was often not the objective of the included studies. Instead, research questions were commonly about the feasibility of new designs or analysis techniques.

To ensure reliable assessment scores, five randomly selected articles were double coded by a second independent rater. Any discrepancies were discussed, and all other quality ratings were then adjusted to ensure consistency among assessments. Afterwards, the percentage agreement was 90.00%, Cohen’s Kappa (κ) was κ = .877 + /-.047, and weighted Cohen’s Kappa (κ_w_) was κ_w_ = .953, thus showing strong agreement between raters (McHugh, 2012).

A summary of the quality assessment of all studies is provided in appendix B to D in the supplementary material. Appendix B shows the quality ratings for fMRI studies, appendix C the one for visual fMEG studies, and appendix D the one for auditory fMEG studies. Individual quality assessment forms can be made available by request.

### 2.5 Statistical analyses

All statistical analyses were carried out in in RStudio using random effect models (see appendix E in supplementary material for used packages) and were between subjects measures, except for the analyses of possible age effects. If a study reported the response rate, latency, or amplitude for different experimental conditions, e. g. long vs. short interstimulus interval (ISI; e. g. Matuz et al., 2012) and/ or for different gestational age (GA) groups (e. g. Eswaran et al., 2021), the study was split into separate groups which represented the response outcomes for one experimental condition and/ or one GA group. 141 groups were identified in this manner, which are subsequently referred to as data groups.

Some studies used longitudinal designs with the same foetuses in multiple data groups, some used cross-sectional designs with different foetuses at different GA. Other studies used a mixture of both of these designs, including longitudinal and cross-sectional assessments in their data groups. Although intended, the dependency between data groups from identical studies was not included as an additional factor in the analyses since the analysis would not be logical due to this heterogeneity.

#### 2.5.1 Removal of noncomparable studies and outliers

The amplitude values from Sheridan et al. (2010) were excluded from all amplitude analyses as this was the only study with negative values (12 data points). The amplitude values from Matuz et al. (2012), Muenssinger et al. (2013b), and Sheridan et al. (2008) were excluded as they calculated the root mean square of the amplitude at peak latency while all other studies reported the average amplitude values without calculating the root mean square. These two different approaches could not be compared to each other, which is why the values of the above-mentioned studies were excluded (48 data points). Although there is an ongoing debate whether outliers should be retained or removed and the appropriateness of various removal techniques (e. g. Aguinis and Gottfredson, 2013; Bakker and Wicherts, 2014; Karch, 2023), in the present study all values above or below two standard deviations (*SD)* from the mean were removed for all continuous variables (272 data points). The rationale for this approach was that these outliers may well be caused by data error such as undetected/ uncorrected movement artefacts and sampling errors. Many studies in this field have not reported (and therefore possibly not assessed) critical demographic information related to the sample, including factors such as maternal health issues or maternal family history of health issues, even though such factors affect general foetal development (e. g. Fleming et al., 2018; Wu et al., 2012) as well as fER (e. g. Doersam et al., 2022; Schleger et al., 2018). These factors could have caused outliers of fER that do not reflect “true” values of the investigated population. For further information on this topic, see Osborne and Overbay (2004) and evidence from Bakker and Wicherts (2014) who showed that the removal of outliers does not increase the *p*-value, the sample size, or reporting errors. 332 data points (0.5% of the dataset) were excluded in total through the removal of noncomparable studies and the removal of outliers.

#### 2.5.2 Descriptive statistics

The range, mean, and *SD* were calculated for all continuous variables and the number per categories was calculated for all categorical variables. Forest plots were created to visualise the range of latency and amplitude values across studies as well as to show the two global averages with their *SD* in relation to the mean and *SD* of individual studies. The studies were then sorted according to their publication year to see how latency and amplitude averages changed over time. In the supplementary material, Figure 6 shows the cumulative forest plot for the standard latency and Figure 7 visualises the one for the standard amplitude.

It was anticipated that technological and methodological advances would lead to a decrease in data artefacts and an increase in accurate signal detection (Anderson and Thomason, 2013). Improved signal-to-noise ratio in both data acquisition and data processing steps was hypothesised to result in more precise estimates with smaller confidence intervals of the investigated outcome variables in more recent, compared to earlier, studies. It was further expected to see a divergence towards standardised ways of presenting and measuring fER, in addition to an increase in reporting of parameters over the years.

#### 2.5.3 Publication and selection bias

Publication and associated biases are related to the “file-drawer problem (Rosenthal et al., 1979). Studies without significant results often fall short of publication which causes a publication bias. Selection bias, on the other hand, refers to a biased selection process of studies, for example by basing the eligibility criteria on the results of specific studies or by using the authors’ social network instead of databases to search for eligible studies (Ahmed et al., 2012). Following recommendations to use multiple tests (e. g. Lin et al., 2018), forest plots, funnel plots, Egger’s regression test (Egger et al., 1997), and the trim-and-fill method (Duval and Tweedie, 2000) were utilised to assess publication and selection bias in the present review. Figures 6-9 in the supplementary material depict possible biases in the present review and meta-analysis.

#### 2.5.4 Moderating analyses

The following continuous variables were analysed for moderating effects on response latency and/ or amplitude values: Publication year, mean GA, stimulus duration, ISI, ratio stimulus duration/ ISI, wavelength, frequency of the standard stimulus, stimulus intensity (light intensity/ sound pressure level (SPL)), number of total presentations (presentations of both standard and deviants), number of standard presentations, mean experimental time, response rate, response latency/ amplitude to the standard, and the study quality. Next, the study identification number, the GA groups, the sample type (atypical vs. typical), the modality (auditory vs. visual), the study design (cross-sectional vs. longitudinal), the stimulus type, the paradigm, and the delivery method were analysed as categorical moderators. Modality and mean GA were also separately added as second level moderators to determine if either of them moderated amplitude and/ or latency values on a higher level.

All moderating analyses were further conducted with the subgroups of data groups that included only typically developing foetuses, only auditory studies, and only visual studies.

The results of these analyses can be found in section 1.3 in the supplementary material.

#### 2.5.5 Correction for multiple comparisons

The *p*-value was set to α = .05 for all analyses. The *p*-value was not corrected for multiple comparisons due to insufficient reporting of methodologies and results in the final dataset that led to a large loss of data groups for most analyses. A corrected *p*-value would have inflated a type II error (Cohen, 1982). This approach should be acknowledged when considering the results.

## 3 Results and discussion

Please see Tables A1 to A5 in the appendix for summaries of the extracted data from the included studies. Table A1 summarises all included fMRI studies, Table A2 and Table A3 show the sample and stimulus characteristics/ the paradigm characteristics and the outcome variables of the included visual fMEG studies. Table A4 and Table A5 illustrate the sample and stimulus characteristics/ the paradigm characteristics and the outcome variables of the included auditory fMEG studies.

It is of note that the sample type “atypical” summarises a variety of studies that investigated samples with the following maternal and foetal risk factors: Gestational diabetes (1 study), maternal family history of diabetes (1 study), maternal eating disorder (1 study), and intrauterine growth restriction (2 studies).

The descriptive statistics and the results of the analyses for the publication and selection biases can be found in the supplementary material.

### 3.1 Main outcome variables

#### 3.1.1 Activated area (fMRI studies)

As summarised in Dunn et al. (2015), auditory stimulation most commonly led to increased activity in the temporal lobe. Between 42.86 % and 100 % of the analysed samples showed activation on the left side (Jardri et al., 2012, Moore et al., 2001), 28.57–100 % had increased activity in the right (Goldberg et al., 2020, Moore et al., 2001), and 46.67–66.67 % had temporal lobe activity with no further laterality information (Fulford et al., 2004, Goldberg et al., 2020, Hykin et al., 1999, Jardri et al., 2008). Goldberg et al. (2020) specified the activation as emerging from Heschl’s gyrus, the location of the primary auditory cortex (e. g. Da Costa et al., 2011; Morosan et al., 2001). Jardri et al. (2012), in contrast, reported a stimulus-specific activation of the lower temporal lobe to the maternal voice in comparison to an unfamiliar female voice and a stimulus-specific activation of the upper temporal lobe to speech in general. Note, however, that this activation pattern was found in only one of their participants at 34 weeks GA and that their sample size was generally very small (*N* = 3). Future studies could explore if there is a stimulus-specific activation pattern within the temporal lobe and at which GA this pattern emerges. Increased activity following auditory stimulation was also found in the left and right middle cingulate, left putamen, and the cortex (Goldberg et al., 2020), as well as in the frontal lobe and sagittal sinus (Moore et al., 2001).

Following visual stimulation, activation was exclusively found in frontal areas (Fulford et al., 2003). As there is just one visual fMRI study, it is not possible to ascertain the effect of stimuli and paradigm characteristics on areas of activation. Since there was only one additional fMRI study compared to Dunn et al. (2015), the current results do not add additional information to the previous findings concerning which brain areas show changed neural activity after external stimulation.

#### 3.1.2 Response rate, response latency, and response amplitude

The response rate had a mean of *M* = 61.74 % in fMRI studies (*SD* = 32.32, range: 0 – 100) which means that, on average, *M* = 61.74 % of the analysed sample showed increased brain activity in at least one brain area after auditory or visual stimulation.

When looking at fMEG studies, the response rate was on average *M* = 68.30 % for the standard stimulus averaged over both simple and discrimination paradigms (*SD* = 20.27, range: 31 – 100) and *M* = 64.75 % for deviant stimuli in discrimination paradigms (*SD* = 6.99, range: 57.00 – 70.59). Discrimination paradigms refer here to fMEG studies that used a standard stimulus and at least one deviant stimulus which were interspersed with silence. Similar to Dunn et al. (2015), who found a similar response rate between fMRI and fMEG studies, the present review shows comparable response rates between fMRI studies and standard stimuli in fMEG studies (*t*(7.90) = -.56, *p* = .593) as well as between standard and deviant stimuli in fMEG studies (*t*(4.48) = .72, *p* = .51).

The response latency (fMEG studies) was on average *M* = 273.97 ms for standard stimuli (*SD* = 75.25, range: 50 – 650 ms) while the data was insufficient to calculate descriptive statistics for deviant stimuli. The standard response latency was not statistically different between auditory and visual studies (*M* = 270.21, *SD* = 54.41 vs. *M* = 284.92, *SD* = 70.47; *t*(67.20) = -1.23, *p* = .224) and was not significantly different between typical and atypical samples (*M* = 274.48, *SD* = 59.28 vs. *M* = 289.33, *SD* = 84.01; *t*(6.32) = -.46, *p* = .66). It is important to note that both fVER and atypical development were investigated to a significantly lesser degree than fAER and typical development. Furthermore, when comparing foetuses with atypical development to their matched controls, the difference was statistically significant with longer response latencies in foetuses with intrauterine growth restriction for both fVER (Morin et al., 2015) and fAER (Kiefer et al., 2008). During an oral glucose tolerance test, foetal responses to auditory stimuli were also significantly delayed compared to healthy controls in mothers with gestational diabetes mellitus (Linder et al., 2015), mothers with a family history of diabetes mellitus (Schleger et al., 2018), and in otherwise healthy, but insulin-resistant mothers (Linder et al., 2014). Only the comparison of foetuses of mothers with and without lifetime anorexia nervosa fell short of significance, which showed, however, descriptively longer response latencies in the group with the maternal risk factor (Doersam et al., 2022).

The mismatch negativity (MMN) was also reported in some of the present studies (Draganova et al., 2005, Draganova et al., 2007, Huotilainen et al., 2005, Muenssinger et al., 2013b, Schleger et al., 2014). The MMN describes an increased negative amplitude in electroencephalographic recordings caused by the presentation of a deviant stimulus among standard stimuli, which shows discriminatory abilities between the two types of stimuli (e. g. Näätänen et al., 1978; Näätänen, 2001). It is an early fronto-central component that usually peaks between 100 – 250 ms after stimulus onset in adult participants (Näätänen, 2001). It can be seen in newborns (e. g. Draganova et al., 2007) and even in premature infants (Cheour-Luhtanen et al., 1996, Tanaka et al., 2001). The MMN had a mean of *M* = 322.18 ms (*SD* = 3.04, range: 318 – 326) in the summarised studies. Several studies also reported the occurrence of an early component without presenting the latency. When considering these studies as well, the MMN was found in 3.28 % of the analysed samples (*SD* = 8.71, range: 0 – 31.58).

Two studies also reported evidence for a late discriminative negativity (LDN; Draganova et al., 2005; Draganova et al., 2007) which occurred within 447 – 470.86 ms after stimulus onset (*M* = 456.69 ms, *SD* = 7.09). This late component of the fER was found in *M* = 10.4% of the analysed samples when studies were included which did not report latency data, but that noted if and how often a late component was present in their data (*SD* = 15.94, range: 0 – 50.00).

None of the more recent studies reported values for MMN or LDN.

As an expansion to the investigations in Dunn et al. (2015), the current review further explored amplitude values of fER. The response amplitude to standard stimuli (fMEG studies) ranged from 4.00 fT to 63.00 fT with a mean of *M* = 25.04 fT (*SD* = 15.4). Again, there was insufficient information to calculate descriptive statistics for deviant stimuli. Likewise to the comparisons for latency values, the amplitude differences of auditory (*M* = 25.53 fT, *SD* = 8.75) vs. visual studies (*M* = 23.95 fT, *SD* = 6.52) were not significant (*t*(109.23) = 1.2, *p* = .234). The comparison between amplitude values in typical (*M* = 25.08 fT, *SD* = 8.42) vs. atypical samples (*M* = 19.6 fT, *SD* = 0) was, however, statistically significant (*t*(130) = 7.45, *p* < .001). Note that only one study that investigated foetuses with risk factors for atypical development reported a mean amplitude. Further studies are required to confirm this finding and to see if the difference between auditory and visual amplitude values reaches significance when more data is available.

Two studies (Draganova et al., 2005, Draganova et al., 2007) also reported amplitude values for the MMN which had a mean of *M* = 19.88 fT (*SD* = .44, range: 19.57 – 20.19).

### 3.2 Age effect

The study data was split into multiple data groups for separate GA groups if authors provided outcome variables for different GA ranges. This was done in 13 studies with the data mostly being separated into three groups with different GA ranges. Five studies reported data for two GA groups and two for twelve GA groups. All other studies reported one global average for their dataset, often including foetuses from a wide GA range. This issue, as well as the variability of how the sample was divided into GA groups, made the analyses of an age effect in the present meta-analysis difficult. This was further complicated by a combination of longitudinal, cross-sectional, and studies evidencing both designs in the dataset. This resulted in a combination of both within and between subjects assessments. 54 data groups did not report any information about age effects. Over half of the fMEG studies, however, investigated an age effect in their own sample and reported whether age moderated fER. In total, 17 fMEG studies reported a significant effect of age on the response rate, latency, or the amplitude of fER while six found no significant age effect in their data.

The nature of the effect was dependent on the variable of interest. With increasing GA, the response rate typically increased (see Table 2), which is contrary to Dunn et al. (2015) who suggested a negative correlation between GA and response rate. Instead, the current findings support a positive correlation. The present result for the relation between GA and response latency is, however, in line with the conclusion of Dunn et al. (2015). As can be seen in Table 2, the response latency decreases with increasing GA. Only two studies investigated an age effect of GA on response amplitude which was not significant in either study (Hartkopf et al., 2016, Muenssinger et al., 2013b). None of the fMRI studies reported age effects on the response rate.

**Table 2:** Age effect on response rate and response latency.

	response rate	response latency
increase	8	0
decrease	1	25
trend decrease	0	4
not significant	6	13
not addressed	118	91

**Note:** The numbers in this table correspond to fMEG data groups, not to individual studies.

The increased response rate with simultaneously decreased latencies in older compared to younger foetuses (see Table 2) can be explained by improved sensory and cognitive development as gestation advances. Improved auditory, visual, and general cognitive abilities heighten the probability of perceiving and processing a sensory stimulus. There is, however, not enough data currently available to investigate if GA also influences the standard response amplitude values, therefore, more studies are needed to answer this question in the future.

### 3.3 Multiple regression analyses

As analysing each possible moderating variable separately inflates the type I error which then leads to false positive effects, separate multiple regression analyses for the response latency and the response amplitude were performed. Afterwards, a stepwise selection method in both directions was conducted based on the Akaike Information Criterion (AIC; Akaike, 1973). This was done to identify the model with the best fit for both outcome variables in each of the three subsamples, namely data groups with typically developing foetuses, auditory data groups, and visual data groups to allow comparisons between them. Data groups with atypical samples could not be analysed separately because of the available data being insufficient to do so.

Please note that the wavelength was not included into those models as the small difference of 5 nm between studies was likely too small to lead to significant different foetal perceptions if the light intensity stayed unchanged. Due to insufficient data for data groups with atypical development, the sample type could also not be included in the analyses for auditory and visual studies.

If values were missing among numerical variables, they were replaced with the corresponding mean using mean imputation (11707 data points, 64.36 % of the data set). A new factor level was created for missing values in categorical variables. This approach minimised the loss of data groups compared to excluding all observations with missing values.

Note that multicollinearity was high among several predictors in the models. It was not possible to reduce the multicollinearity without removing possible meaningful variables or factor levels. A more thorough description for each analysis of the three subsamples can be found in the supplementary material.

#### 3.3.1 Response latency

When all continuous variables (publication year, mean GA, stimulus duration, ISI, ratio stimulus duration/ ISI, light intensity, frequency and SPL of the standard stimulus, the number of total presentations, the number of standard presentations, the mean experimental time, the response rate, latency, and amplitude of the standard stimulus, study quality) and all categorical variables (study identification number, GA groups, modality, study design, stimulus type, paradigm, delivery method) were included in the multiple regression analysis, none of those variables significantly predicted the response latency for either subsample (typical samples: all *p* ≥ .152, auditory studies: all *p* ≥ .127, visual studies: all *p* ≥ .104).

#### 3.3.2 Response amplitude

In typical samples, the overall model was significant (*F*(94, 36) = 3.81, *p* < .001; R² = .91; adjusted R² = .67) with the frequency (Hz) of the standard (β = 0.24, *p* < .001), the response rate (β = 0.22, *p* = .01), and several GA groups (0.10 < β < 9.98, <.001 < *p* < .047) being significant predictors of the response amplitude.

The overall model was also significant for auditory studies (*F*(80, 16) = 1037, *p* < .001; R² = .99; adjusted R² = .99). Significant predictors were the mean GA (β = 1.23, *p* < .001), the frequency (Hz) of the standard (β = 0.24, *p* < .001), and the study quality (β = −114.9, *p* = 0.31). Several GA groups (-1.78 < β < 61.46, <.001 < *p* < .009) and some individual studies (44.43 < β < 333.6, <.011 < *p* < .041) were also significant.

In visual studies, the overall model was again significant (*F*(32, 11) = 10.56, *p* < .001; R² = .97; adjusted R² = .88). Significant predictors were the ISI (β = −0.01, *p* = .002), the ratio of stimulus duration to ISI (β = −119.4, *p* < .001), and the mean experimental time (β = 2.4, *p* = .017). Eswaran et al. (2005); β = -9.12, *p* = 0.044) and several GA groups (7.76 < β < 32.98, <.001 < *p* < .023) were further significant.

It is of note that in each of the analyses multiple coefficients were excluded from the model due to singularities (typical: 62; auditory: 48; visual: 29). The singularity was likely caused by multicollinearity between variables and/ or a lack of variability within the sample (Hill and Adkins, 2001). This can be explained by the relatively small number of data groups available for the amplitude analyses and the chosen method of mean imputation for missing values (Fichman and Cummings, 2003). There were further age-specific effects in all three analyses as indicated by multiple significant factor levels of the coefficient GA group, which highlights the influence of GA on the amplitude of fER. The study-specific effects in the models for the auditory and visual subsamples could imply the presence of further methodological differences between studies which went beyond the sample, stimulus, and paradigm characteristics that had been extracted for this review. Those might be subject to future analyses.

### 3.4 Moderating analyses

Due to the large amount of missing information in the dataset, each of the moderating variables was also analysed separately to see if there were any trends that fell short of significance in the multiple regression analyses. In contrast to the previous analyses, missing values were excluded in all of those analyses. The results of the moderating analyses fell short of significance when correcting for α-inflation due to multiple tests. When looking at the uncorrected results, which are described in more detail in the supplementary material, the light intensity, the number of presentations, and the stimulus type are the three most significant moderators for fVER. All of those had been previously suggested as being relevant moderators of fVER latencies (Dunn et al., 2015).

The results of the uncorrected moderating analyses for fAER show SPL as the most significant moderator for response latencies. The ratio of stimulus duration/ ISI, the number of presentations, and the mean experimental time were all not significant, therefore not supporting the assumption of Dunn et al. (2015) regarding moderating effects of those variables on fAER latencies. Instead, the stimulus type was the most significant variable for response latencies in typically developing foetuses. The ratio of stimulus duration/ ISI, the number of presentations, and the mean experimental time were, however, significant moderators of the response amplitude of fVER. The ISI and the stimulus type significantly moderated the amplitude values of fAER. There was further additional variability in amplitude values between individual studies which was unrelated to any of the analysed stimulus and paradigm characteristics (see full results in supplementary material).

### 3.5 Limitations and recommendations

#### 3.5.1 Limitations and recommendations for future studies

Over the last decade, only twelve new studies met the present eligibility criteria and only 46 studies were generally included in the present review and meta-analysis. This shows that the field of fER is still small and that there is need for further studies to better understand how foetuses perceive the world around them. Future fMRI studies will help to confirm the present findings and will locate neural activity more precisely within the foetal brain. This is especially important for fVER as the quality of the included fMRI studies was rated either “sufficient” or “insufficient” with none in the “good” or “very good” category (see supplementray material).

Given that most studies did not report important information about several sample, stimulus, and paradigm characteristics, many data groups could not be included in various individual analyses. This decreased the overall power for statistical analyses, which also limited the present multiple regression and moderating analyses, especially when considering the weak publication bias that was found (see supplementray material). To allow a more comprehensive understanding of which variables affect fER, authors should provide all necessary information and are strongly encouraged to make their raw data available via open access to improve replicability and to facilitate future reviews and meta-analyses on this topic. To further enhance the reliability of study results, authors should report how many trials (per participant) were included in their analyses and report all relevant outcome variables, namely response rate, response latency, response amplitude, and/ or regions with changed brain activity to facilitate comparisons between studies.

Many researchers did not report the GA which did not allow a comprehensive understanding of their results when considering the evidence for age related changes in response rates and latencies of fER (see paragraph 3.3). If authors analyse age effects in their own data and report the results irrespective of whether they are significant or not for all outcome variables in the future, then this will improve our understanding of how fER change with increasing GA. Expanding the investigated GA to earlier age ranges will be helpful to determine at which GA auditory and visual stimuli begin to elicit fER. Knowing when and how typically developing foetuses normally respond to different sensory stimuli and how fER change as gestation progresses can offer valuable information about foetal development which can be used for prenatal clinical assessments. This knowledge could further improve existing early interventions and aid the development of new ones. Those might offer foetuses with risk factors for atypical development and foetuses that already show disadvantageous developmental trajectories a better chance of receiving the necessary support or treatment to enable a more favourable development.

It is highly recommended that authors of future studies should follow more standardised protocols for data collection and data analyses to enhance the comparability between studies and the possibility of successfully measuring fER. For example, standardising the time frames for visual inspection of fER and the standardising processes for averaging data over multiple trials and participants will facilitate future comparisons. Once a basic understanding of foetal sensory abilities has been achieved by synthesising the results from standardised protocols, study designs could then start to diversify once more. This would enable the field to explore which stimulus characteristics most reliably elicit a fER, e. g. which light intensity is best suited to induce fVER, how often a stimulus can be presented before it no longer elicits a fER, and how bimodal stimulation affects foetal brain responses.

To allow direct comparisons between fMRI and fMEG studies, stimuli could be more aligned across measures. Simple stimuli could offer the benefit of reducing the higher attrition rate in fMRI studies, which could be based on higher stimulus complexity leading to lower response rates, particularly earlier in gestation.

Should future studies implement the above-mentioned recommendations, then the study quality will likely improve across the field, with more ratings in the “good” to “very good” range. The quality assessment form that has been created for this review (see appendix A of supplementary material) could aid authors of future studies by providing a basis for which information should be included in publications to allow comparisons of results across studies.

#### 3.5.2 Limitations of the present review

The current review did not correct for multiple comparisons and subsample analyses in order to reduce the type II error (Cohen, 1982). Further, the stepwise selection method was based on the AIC which is only one of several possibilities. The AIC was chosen as the present work was the first to analyse the value of various sample, stimulus, and paradigm characteristics of foetal fMRI and fMEG studies for the prediction of fER. The AIC is known to outperform other information criteria in exploratory analyses where the focus is on identifying relevant variables rather than finding the most parsimonious model (Zhang et al., 2023). Furthermore, the AIC is better suited for complex models with many possible predictors (Vrieze, 2012). The AIC may lead to over-selection of variables (Zhang et al., 2023), although it should be noted that the present work was unlikely to find the “true” model of effects on fER given that it is the first to use a multiple regression analysis for the field of foetal sensory fMRI and fMEG studies. Rather, the results are to be seen as a guide for future studies to identify which sample, stimulus, and paradigm characteristics are important to consider when conducting a study to investigate fER after sensory stimulation and how these variables might influence the results of the study. Future meta-analyses might consider using a different set of criteria to guide the selection method. This is also true for how to deal with missing information. In the multiple regression analyses, missing numerical values were replaced by mean values while missing categorical values were summarised in a separate factor level to minimise the exclusion of data given that the dataset contained a substantial number of missing values. Even though there are several statistically more sophisticated approaches for dealing with missing values, such as multiple imputation and maximum likelihood estimation (e. g. Enders, 2013), the data set for the present study contained too many missing values in both the numerical predictors as well as in the outcome variables (64.36%) to enable the application of those approaches. Mean imputation was therefore chosen to allow exploratory analyses of the available data to identify relevant predictors of latency and amplitude values. If future studies provide more comprehensive information, future meta-analyses will have fewer missing values in their dataset which will then allow them to handle missing data more appropriately.

Many studies did not report a mean GA which limited the range of possible analyses in this area. To keep the loss of data groups as low as possible, missing mean GA values were replaced by the mean of GA ranges when those were reported. This approach may have decreased the accuracy of the mean GA that was used to perform the analyses by adding variability through how it was calculated (reported mean GA vs. calculated mean of reported GA range). This issue could be eliminated in future work if researchers report the mean GA with *SD* and/ or make raw data with the GA of all participants available via open access.

The group “atypical samples” included a small number of studies with a variety of maternal and foetal risk factors, such as gestational diabetes and intrauterine growth restriction. Ideally it would be better to analyse the effect of different risk factors for atypical development separately in the future. This was not possible in the present review due to insufficient power, given the small number of included studies with foetal and maternal risk factors (*n* = 5). Instead, the studies were grouped together as atypical samples to see if there is a general trend for delayed responses in foetuses with risk factors for atypical development. This was based on the finding that three of the five atypical samples showed larger response latencies when compared to their matched controls (Doersam et al., 2022, Kiefer et al., 2008, Morin et al., 2015).

Finally, inconsistencies were present in the current review between the statistical approaches used to test the existence of a publication and/ or selection bias (see supplementary material). While some of the tests supported the presence of a publication bias, others did not. Future reviews and meta-analyses should follow the current approach by using more than one of these tests and should analyse the quality of individual studies to assess if - and to which extent - biases might be present in the data.

## 4 Conclusion

The present review and meta-analysis adds to the body of research showing that foetuses can perceive and process auditory as well as visual stimuli, certainly in the last trimester of pregnancy (e. g. Dunn and Reid, 2020, Lecanuet and Schaal, 1996). In line with Dunn et al. (2015), the summarised fMRI studies demonstrate that auditory stimuli primarily activate areas in the temporal lobes with additional activation in various other areas that are distributed over the foetal brain. Visual stimuli activate only frontal regions.

The present review further confirms that response latencies and amplitude values of fER can be successfully measured with fMEG and that they are comparable between auditory and visual modalities. There was, however, a slight trend for shorter latencies and larger amplitude values in auditory compared to visual studies. In addition, foetuses with risk factors for atypical development responded significantly slower compared to foetuses with typical developmental trajectories when contrasted to matched controls.

It is important to note that 63.04 % of the included studies had already been summarised by Dunn et al. (2015). The effect of the methodological differences in the field has, however, only now been analysed via a meta-analytical approach. This allows for the quantification of possiblemoderating effects of sample, stimulus, and paradigm characteristics on response rate, response latency, and amplitude values, which is in contrast to the qualitative work of Dunn et al. (2015). The meta-analysis shows that fER were moderated by several stimulus and paradigm characteristics which appear to have an influence that is modality specific. The uncorrected moderating analyses confirm the assumption of Dunn et al. (2015) whereby light intensity, number of presentations, and stimulus type are significant moderators of latencies of fVER. The previous assumption of Dunn et al. (2015) for fAER was not supported. Instead, the SPL was the most significant moderator for auditory response latencies. The amplitude analyses showed several significant moderators for fER, with ISI and publication year being the most significant moderators for both fAER and fVER. This highlights the importance of the time in between stimulus presentations to potentially allow receptor recovery as well as the significance of the influence of technological advances over the last 40 years in the field of fER. Due to insufficient data regarding the GA, it remains inconclusive how GA affects amplitude values. However, older foetuses tend to show higher response rates and shorter latencies compared to younger foetuses.

After achieving a better knowledge of unimodal processing in the foetal brain and after gaining a basic understanding of foetal sensory abilities in general, implementing more bimodal stimulation paradigms and investigating foetal gustatory, olfactory, and tactile evoked responses will likely feature in future work to allow a more comprehensive insight in how (healthy) foetuses experience and perceive the world around them. This is particularly important as, under normal circumstances, the foetus is exposed to stimulation from more than one modality at a time (Partanen and Virtala, 2017). Furthermore, perceptual processing can happen simultaneously in more than one modality, for example, through modality independent variations in sensory information such as duration and intensity, which lead to intersensory facilitation (e. g. Bahrick et al., 2006; Lickliter et al., 2017).

If we understand how typically developing foetuses respond to certain stimuli at a specific GA, we can then look at foetuses with atypical development. Future work will be able to determine the developmental impact of certain risk factors with clinical assessment tools based on studies that investigated sensory and general cognitive abilities of the foetus. This knowledge can then provide the framework for the development of targeted interventions for foetuses at risk for atypical development.

Taken together, there remains significant methodological variability between studies of fER with fundamental differences between fMRI and fMEG studies in regard to sample size, GA range, and stimulus complexity. The need for standardised protocols, larger sample sizes, and a more comprehensive description of the methods and results are therefore necessary for the improvement of work in this domain. By implementing the recommendations outlined in this paper, future studies will not only improve their own quality but they will also facilitate comparisons between each other. This will enrich the field and enable a strong foundation for understanding how auditory and visual stimuli are processed in the foetal brain.

## Data statement

The data that support the findings of the present review and meta-analysis are openly available in OSF at https://doi.org/10.17605/OSF.IO/CXRYF.

## CRediT authorship contribution statement

**Leonie Loehn:** Writing – original draft, Writing – review & editing, Visualization, Validation, Software, Resources, Project administration, Methodology, Investigation, Funding acquisition, Formal analysis, Data curation, Conceptualization. **Kirsty Dunn:** Writing – review & editing, Supervision, Resources, Methodology, Conceptualization. **Michelle To:** Writing – review & editing, Supervision, Conceptualization. **Vincent M. Reid:** Writing – review & editing, Supervision, Resources, Methodology, Funding acquisition, Conceptualization.

## Declaration of Competing Interest

The authors declare that they have no known competing financial interests or personal relationships that could have appeared to influence the work reported in this paper.

## Figures and Tables

**Table A1:** Extracted data from included fMRI studies

identification		sample characteristics				stimulus characteristics						paradigm characteristics				outcome variables		
authors	date	recruited sample	ana-lysed sample	attri- tion rate (%)	GA (weeks)	stimulus type	stimulus duration (ms)	ISI (ms)	ratio stimulus duration /ISI	frequency (Hz)/ wave- length (nm)	intensity (dB/ lux)	para-digm	delivery method	number of presen-tations	mean exp. time (min)	number of trials included in analyses	response rate (%)	activated area

Fulford et al.	2004	17	15	**11.76**	≥ 36	vibroacoustic stimulation	4000	1600	**1:4**	NA	95–100 dB	block design	acoustic stimulator	30	**10**	NA	**46.67**	temporal lobe
Goldberg et al.	2020	9	8	**11.11**	33–38 36.14 + /- 1.4	maternal voice (singing a lullaby)	15,000	10,000	**1:0.67**	NA	NA	block design	mother singing	9	3.73	NA	**100** **75** **NA**	right Heschl's gyrus left Heschl's gyrus right & left middle cingulate cortex, left putamen
Hykin et al.	1999	4	3	**25**	38.00 – 39.14	recorded maternal voice (nursery rhyme)	15,000	15,000	**1:1**	NA	100 dB	block design	speaker & pipe	18	**9**	NA	**66.67**	temporal lobe
Jardri et al.	2012	6	3	**50**	33–34	recorded maternal/ unfamiliar female voice, pure tones	21,000	12,000	**1:0.57**	500, 700, & 900 Hz	100 dB	block design	headphones on abdomen	9	**14.85**	NA	**100** 33.33 (34 weeks GA)	left temporal lobe specific sensitivity to speech/ maternal voice in upper/ lower temporal lobe
Jardri et al.	2008	6	3	**50**	28–33	pure tones	21,000	12,000	**1:0.57**	500, 700, & 900 Hz	100 dB	block design	headphones on abdomen	12	**6.6**	NA	**66.67**	left temporal lobe
Moore et al.	2001	16 exp.: 12 control: 4	7	**41.67**	37–41	recorded Spanish guitar music	15,000	15,000	**1:1**	NA	85 dB	block design	headphones on abdomen (exp.)/ mother’s ears (control)	30	**15**	exp: 62 (6–14 per subject) control: 30 (5–19 per subject)	**71.43** 42.86 28.57 14.29 28.57	for at least one area left temporal lobe right temporal lobe frontal lobe sagittal sinus
Fulford et al.	2003	12	8	**33.33**	≥ 36	LED cluster	8000	16,000	**1:2**	red	1100–1200 lux	block design	light tube	40	**16**	**124**	**62.5**	frontal region

**Note:** Items in bold have been calculated by the current authors. GA = gestational age, ISI = interstimulus interval, NA = not available, exp. = experimental, block design = stimulation vs. silence. None of the fMRI studies investigated age effects.

**Table A2:** Extracted data from visual fMEG studies – sample and stimulus characteristics

identification		sample characteristics				stimulus characteristics					
authors	date	recruited sample	analysed sample	attrition rate (%)	GA (weeks)	stimulus type	stimulus duration (ms)	ISI (ms)	ratio stimulus duration/ ISI	wavelength (nm)	intensity (lux)
Bisgin et al.	2019 (1)	36	30	**16.67**	27–38	NA	NA	NA	NA	NA	NA
Eswaran et al.	2021 (1)	158	112	**29.11**	33.27 + /- 2.66	NA	500	2500–3500	**1:5–1:7**	630	**∼4000**
Eswaran et al.	2004	12 recordings 28–32 GA: 10 33–36 GA: 14 37–39 GA: 7	9 recordings 28–32 GA: 5 33–36 GA: 6 37–39 GA: 1	**25**	28–39	flash	500	**1500**	**1:3**	625	8800
Eswaran et al.	2005 (1)	11	11	**0**	27–36	flash	33	2000	**1:60.61**	625	8800
Eswaran et al.	2002b	17	10	**41.18**	28–36	flash	33	2000	**1:60.61**	625	8800
Matuz et al.	2012 (1)	40 recordings long: 26 short: 14	37 recordings long: 23 short: 14	**7.50** long: **11.54** short: **0**	30–38	flash set^a^	500	2000 long: 12,500 short: 4500	**1:4**	630	**∼4000**
McCubbin et al.	2007	27	25	**7.41**	30–36	flash	500	3500	**1:7**	630	**∼4000**
Morin et al.	2015	128	**74** IUGR: 14 SGA: 23 control: IUGRc: 14 SGAc: 23	**42.19**	29.00–39.43, 34.29 IUGR & IUGRc: 32.86 SGA & SGAc: 34.0	flash	500	2000 + /- 500	**1:3–1:5**	625	8000
Sheridan et al.	2008	25	25	**0**	29–37	flash set^a^	500	200 ITI: 10,000	**1:0.4**	630	**∼4000**

**Note:** Items in bold have been calculated by the current authors. GA = gestational age, ISI = interstimulus interval, NA = not available, long = long intertrial interval, short = short intertrial interval, ITI = intertrial interval, IUGR = intrauterine growth restriction, SGA = small for GA. ^a^ Left as separate stimuli as foetal visual evoked responses to all individual flashes were reported.

**Table A3:** Extracted data from visual fMEG studies – paradigm characteristics and outcome variables

Identification		paradigm characteristics				outcome variables				
authors	date	paradigm	delivery method	number of presentations	mean exp. time (min)	number of trials included in analyses	response rate (%)	response latency (ms)	response amplitude (fT)	sig age effect?
Bisgin et al.	2019 (1)	NA	NA	NA	NA	172	**81.25**	273 + /-81	> 5*	higher response rate ≥ 35 vs. < 35 weeks GA
Eswaran et al.	2021 (1)	simple	fiber-optic cable/ woven panel	**∼103**	6	237	69.20	293.7 + /- 114.5	> 5*	n. s.: 27–39 weeks GA: -1.35 ms/ week
Eswaran et al.	2004	simple	fiber-optic cable	180	6	NA	28–32: 60 33–36: 7**1.43** 37–39: 28**.57**	fP200/ fP300 28–32: 248.6/ 366 33–36: 196.17/ 340 37–39: 115/ 368	fP200: **17.45 + /- 11.15** fP300: **18.78 + /- 10.19**	for fP200 (*r* = -.79, *p* < .05)
Eswaran et al.	2005 (1)	simple	fiber-optic cable	**177**	6	NA	68	251.54 **+ /- 83.58**	26	no
Eswaran et al.	2002b	simple	fiber-optic cable	180	6**.1**	NA	NA	180–390	15–30	latency decrease with increasing GA
Matuz et al. ^a^	2012 (1)	simple^x^	fiber-optic cable/ woven panel	360	18.75–30.75	minimum: 70	**37.23** long: 45.4**5** short: 23.8**1**	long: **418.07 + /- 138.96** short: **341.6 + /- 145.21**	long: 8.2**8 + /- 1.75** short: **6.84 + /- 1.62**	more bimodal responses in older foetuses
McCubbin et al.	2007	simple	fiber-optic cable/ woven panel	150	8	NA	**92.59**	180–770	not addressed	inconclusive
Morin et al.	2015	simple	fiber optic wires/ light pad	**240**	10	NA	NA	IUGR: 233.1**5 + /- 58.69** IUGRc: 184.**55 + /- 51.25** SGA: 216.**08 + /- 62.54** SGAc: 219.9 **+ /- 58.81**	> 4*	not addressed
Sheridan et al.^a^	2008	simple	fiber-optic cable/ woven panel	360	30	NA	**28.95**	35**3.36 + /- 109.29**	> 8* 15.35	not addressed

**Note:** Items in bold have been calculated by the current authors. GA = gestational age, ISI = interstimulus interval, NA = not available, long = long intertrial interval, short = short intertrial interval, IUGR = intrauterine growth restriction, IUGRc = control group for IUGR, SGA = small for GA, SGAc = control group for SGA. ^a^ Amplitude values were excluded from statistical analyses as only root mean squares were reported. ^x^ Tone was inserted between trials for response recovery to visual stimuli; foetal auditory and foetal visual evoked responses are reported separately. * Criterion for foetal visual evoked response.

**Table A4:** Extracted data from auditory fMEG studies – sample and stimulus characteristics

identification		sample characteristics				stimulus characteristics					
authors	date	recruited sample	analysed sample	attrition rate (%)	GA (weeks)	stimulus type	stimulus duration (ms)	ISI (ms)	ratio stimulus duration/ ISI	frequency (Hz)	intensity (dB)
Bisgin et al.	2019 (2)	36	**32**	**11.11**	27–38	pure tones	NA	NA	NA	NA	NA
Doersam et al.	2022	43 subsamples: NA	23 maternal eating disorder (mED): 12 control: 11	**46.51**	27–37 mED: 30.42 + /- 2.75 control: 31.45 + /- 3.21	pure tones	500	1500	**1:3**	500 (75%) 750 (25%)	95
Draganova et al.	2005	12	12	**0**	33–36	tone bursts	100	condition 1: 500–1100 condition 2: 800	500: **1:5** 1100: **1:11** 800: **1:8**	500 (88%) 750 (12%)	110
Draganova et al.	2007	18	18	**0**	28–39	tone bursts	100	700–900	**1:7–1:9**	500 (88%) 750 (12%)	120
Draganova et al.	2018	55	38	**30.91**	31–40	amplitude modulated tones (AM)	AM 2 & 4: 10,000 AM 8: 5000 AM 27–42: 1000 AM 78–91: 1000	AM 2 & 4: 3000 AM 8: 3000 AM 27–42: 3000–3500 AM 78–91: 4000–4500	AM 2 & 4: **1:0.3** AM 8: **1:0.6** AM 27–42: **1:3–1:3.5** AM 78–91: **1:4–1:4.5**	500 AM 2, 4, 8, 27–42, 78–91	AM 2–8: 78 AM 27–91: 86
Eswaran et al.	2021 (2)	158	146	**7.59**	33.17 + /- 2.59	tone bursts	100	3000 + /- 500	**1:25–1:35**	500 (80%) 1000 (20%)	110
Eswaran et al.	2005 (2)	11	11	**0**	27–38	tone bursts	1000	2000 + /- 500	**1:1.5–1:2.5**	500 (80%) 1000 (20%)	120
Eswaran et al.	2002a	10	T1: 10 T2: 5	T1: **0** T2: **50**	T1: 30–35 T2: 36–40	tone bursts	100	1000 + /- 100	**1:9–1:11**	500 (80%) 1000 (20%)	120
Hartkopf et al.	2016	30 (60 datasets)	(42 datasets)	(**30** datasets)	28–39 32.86 + /- 3.12	sequence of 8 syllables^a^	500	500 between sequences (ITI): 4000–5000	**1:1**	/ba/: 500–750 (50%) /bi/: 200–280 (50%)	95
Holst et al.	2005	18	16	**11.11**	27–39	pure tones	500	2000	**1:4**	500 (80%) 700 or 1000 (20%)	120
Huotilainen et al.	2004	17	17	**0**	35–40 37.71	tone bursts	100	700	**1:8**	500 (80%) 1000 (20%)	85–90
Kiefer et al.	2008	hypotrophic: 14 (asymmetric: 10, symmetric: 4) control: 15	hypotrophic: 14 (asymmetric: 10, symmetric: 4) control: 15	**0**	hypotrophic: 27–38, 33 asymmetric: 7–38, 37 symmetric: 31–33, 32 control: 27–39, 33	pure tones	500	2000 + /- 500	**1:3–1:5**	500 (80%) 700 (20%)	120
Lengle et al.	2001	19	11	**42.11**	29–40	tone bursts	50	1250 (+/- jitter)	**1:25**	1500	100
Linder et al.	2014	22	13	**40.91**	27–36, 30.9	pure tones	500	1500	**1:3**	500 (75%) 750 (25%)	95
Linder et al.	2015	63 gestational diabetes (GD): 15 control: 48	40 GD: 12 control: 28	**36.51** GD: **20.00** control: **41.67**	27 – 36, 30.3 + /- 2.5 GD: 30.1 + /- 2.6 control: 30.9 + /- 2.7	pure tones	500	1500	**1:3**	500	95
Matuz et al.	2012 (2)	long: 26 short: 14/26 recordings: 42	long: 6 short: 5/ 14 recordings: 6	long: **76.92** short: **64.29** recordings: **85.71**	30–38	pure tones	500	long: 20,000 short: 12,000	long: **1:4** short: **1:2.4**	500	110
Moser et al.	2021	60	56	**6.67** recordings: **22.86**	25–40 analysed sample: 32.96 + /- 4.08	sequence of 4 pure tones	tone: 200 sequence: 2000	between tones: 400 between sequences (ITI): 1700	tones: **1:2** sequence: **1:0.85**	500 (25 – 75%) 750 (25 – 75%)	90
Muenssinger et al.	2013a (1)	25	14	**44**	30–38 analysed sample: 34.57 + /- 2.34	pure tones & white noise (WN)	tones: 500 WN: 1000	2000–2500	tones: **1:4, 1:5** WN: **1:2, 1:5**	tones: 500, 750 WN: 80–1000	95
Muenssinger et al.	2013a (2)	12	5–10	**16.67–58.33**	25.7 + /- 2.4	tone bursts, WN, & attenuated WN	100	1000	**1:10**	tone: 500 WN & attenuated WN: NA	95
Muenssinger et al.	2013b	41	36	**12.2**	analysed sample: 30–39, 34.83 + /- 2.93	sequence of 8 tone bursts^a^	tone: 70 sequence: 2660	between tones: 300 between sequences (ITI): 4000–5000	tone: **1:0.23** sequence: **∼1:1.5-∼1:1.88**	500 (87.5%) 750 (12.5%)	95
Porcaro et al.	2006	12	12	**0**	36–40	pure tones maternal heartbeat (mHB)	500	2631	**1:5.262**	tone: 1000 (*n* = 10) 500 (*n* = 2) mHB: NA	103
Preissl et al.	2001 (1)	22	22	**0**	30–40, **36.8 + /- 2.56**	tone bursts	100	2000 + /- 100	**1:19–1:21**	1000	120
Schleger et al.	2014	30	23	**23.33**	30–39 analysed sample: 34.9 + /- 2.8	sequence of 2 (80%) vs. 4 tone bursts (20%)	tone: 70 sequence: 2 tones: 290 4 tones: 590	between tones: 150 between sequences (ITI): 1000	tone: **1:2** sequence: 2 tones: **∼1:3.45** 4 tones: **∼1:1.69**	500	95
Schleger et al.	2018	167 subsamples: NA	52 maternal family history of diabetes: 15 control: 37	all: 68.86 subsamples: NA	28–36, 30 + /- 2	pure tones	500	1500	**1:3**	500	95
Schleussner & Schneider	2004	38	12	**68.42**	27–40	tone bursts	50	1000 + /- 200	**1:16–1:24**	500	100
Schleussner et al.	2004	NA	38	NA	27–39	tone bursts	50	1000 + /- 200	**1:16–1:24**	500	100
Schleussner et al.	2001	27 recordings: 64	NA	NA	29–40	tone bursts	50	recording 1: 1000 recording 2: 800–1200	800: **1:16** 1000: **1:20** 1200: **1:24**	500	100
Schneider et al.	2001	27 recordings: 64	21	**22.22**	29–41	tone bursts	50	recording 1: 800–1200 recording 2: 1000	800: **1:16** 1000: **1:20** 1200: **1:24**	500	100
Sheridan et al.	2010	22	20	**10**	29–38	sequence of 2 tone bursts	tone: 70 sequence long: 440 short: 210	between tones: short: 70 long: 300 between sequences (ITI): long: 700 short: 705	tone short: **1:1** long: **1:4** sequence long: ∼1:1.59 short: ∼ 1:3.36	500 followed by 500 (85%) or 750 (15%)	120
Zappasodi et al.	2001	12	12	**0**	36–40	pure tones	500	2131	**1:4.26**	500 1000 *(analysed together)*	103

**Note:** Items in bold have been calculated by the current authors. GA = gestational age, ISI = interstimulus interval, NA = not available, long = long intertrial interval, short = short intertrial interval, ITI = intertrial interval. ^a^ Left as separate stimuli because foetal auditory evoked responses to all individual tones were reported.

**Table A5:** Extracted data from auditory fMEG studies – paradigm characteristics and outcome variables

identification		paradigm characteristics				outcome variables				
authors	date	paradigm	delivery method	number of presentations	mean exp. time (min)	analysed trials	response rate (%)	response latency (ms)	response amplitude (fT)	sig age effect?
Bisgin et al.	2019 (2)	NA	NA	NA	NA	133	**60**	252 + /- 75	> 5*	response rate: n. s.
Doersam et al.	2022	discrimination^z^	tube/ balloon	**300**	10	NA	NA	mED: 212 + /- 68.7 control: 168.5 + /- 70.4	not addressed	not addressed
Draganova et al.	2005	discrimination	tube/ bag	**∼1067**	16	S: 300 D: 44	60	S: **259.27 + /- 63.05** D1: **321.24 + /- 38.62** D2: **468.14 + /- 38.2** MMN: **318.35 + /- 36.99** LDN: **470.86 + /- 32.17**	S: < 10 D: > 20	not addressed
Draganova et al.	2007	discrimination	tube/ bag	S: ∼600 D: 70	12	S: 600 D: 70	STD: 56 early: 32 late: 32 MMN: 46 LDN: 39	S (early)**: 222 + /- 40.75** S (late): 380 + /- 52 MMN: 322.25 + /- 41.75 LDN: 455 + /- 41	S: 7–12 D: > 20	no
Draganova et al.	2018	discrimination	tube/ balloon	per trial AM 2 & 4: 38–46 AM 8: 65–75 AM 27 & 42: 75–86 AM 78 & 91: 55–66	**40**	NA	AM 2 Hz: 70 AM 4 Hz: 57 AM 8 Hz: 55 AM 27 Hz: 86 AM 42 & 78 Hz: 50 Am 91 Hz: 31	AM 2 Hz: 397.25 + /- 106 AM 4 Hz: 367.13 + /- 82 AM 8 Hz: 301.83 + /- 41 AM 27 Hz: 150.33 + /- 33 AM 42 Hz: 184.29 + /- 28 AM 78 Hz: 170.29 + /- 42 AM 91 Hz: 173.25 + /-26	not addressed	31–39 weeks GA: latency decrease for AM 4 Hz (*R*^2^ = 0.283, *p* = 0.017)
Eswaran et al.	2021 (2)	discrimination^z^	tube/ bag	**∼116**	6	321	73.83	290.7 + /- 125.5	> 5*	n. s.: −1.97 ms/ week at 28–38 weeks GA
Eswaran et al.	2005 (2)	discrimination^z^	tube/ bag	120 S: 96 D: 24	6	NA	61.76	264.6	S: 30.1	not addressed
Eswaran et al.	2002a	discrimination^z^	tube/ bag	NA	NA	NA	T1 & T2: 80 each	**260.25 + /- 56.11**	**13.84 + /- 5.61**	not addressed
Hartkopf et al.	2016	discrimination	tube/ balloon	560 each (1120 in total) S: 980 D: 140	30	NA	NA	not addressed	average normalised mean (/ba/ vs. /bi/: n. s.): S: **1.035 + /-.31** D: **1.105 + /-.53**	n. s. (*p* = 0.540)
Holst et al.	2005	discrimination^z^	tube/ balloon	**144** S: **∼115** D: **∼29**	**6**	NA	83	27–31: 288 + /- 90.5 32–35: 251 + /- 86.0 36–39: 197 + /- 50.7	> 7* 23.4	-9.6 ms/ week
Huotilainen et al.	2004	discrimination	tube/ funnel	NA	NA	NA	S: **41.18** D: **70.59**	S: 230.43 **+ /- 145.71** MMN: **325.65 + /- 90.45**	29.**19 + /- 9.42** S: **12.01 + /- 7.82** MMN: **20.19 + /- 10.14**	not addressed
Kiefer et al.	2008	discrimination^z^	tube/ balloon	**∼144–192** S: **∼115–154** D: **∼29–38**	6–8	NA	atypical: 72.73 control: 85.11	hypotrophic: 366.7 + /- 27 asymmetric: 349.4 + /- 32 symmetric: 413 + /- 54 control: 293.2 + /- 19 *(adjusted for mean GA)*	hypotrophic: 19.6 control: 22.2	-12.74 ms/ week data refitted for birth outcomes: −12.39 ms/ week (*p* = .0047) atypical vs. control: + 73.5 ms (*p* = .034) asymmetric: n. s.: + 57.6 ms symmetric: + 120.0 ms (*p* = .045)
Lengle et al.	2001	simple	tube	**384–768**	**8.32–16.64**	NA	53.57	**243.2 + /- 24.49**	**11.89 + /- 3.48**	latency decrease with increasing GA
Linder et al.	2014	discrimination^z^	tube/ bag	**180** S: **135** D: **45**	6	NA	NA	297 + /- 99	not addressed	not addressed
Linder et al.	2015	simple	tube/ bag	**180** S: **135** D: **45**	6	NA	NA	GD: NA control: 260 + /- 90	not addressed	not addressed
Matuz et al.	2012 (2)	^x^	tube/ bag	**90**	**18.75–30.75**	NA	NA	long (*n* = 14): **317.67** **+ /- 133.8** short (*n* = 5): n.s.	long (*n* = 14): **9.07** **+ /- 3.44** short (*n* = 5): n.s.	more bimodal responses in older foetuses
Moser et al.	2021	learning phase: simple/ discrimination test phase: discrimination	tube/ balloon	2x learning phase: 120 (90/ 120 x S, 30/ 0 x D) 1 x test phase: 720 (540 x S, 180 x D)	26	46 trials per condition	participants**: 76.79** recordings: **77.14**	early: 50–350 late: 350–650	NA	for sssD, rest n. s. 25–34 weeks GA: sig. response increase from T3 to T4 (*t*(29) = 2.8, *p* = 0.009) 35–40 weeks GA: sig. decrease from T3 to T4 (*t*(25) = -2.14, *p* = 0.042) sig. neg. correlation T4-T3 (*r*(62) = -0.319, *p* = 0.01)
Muenssinger et al.	2013a (1)	discrimination	tube/ balloon	70 per condition (210 in total)	8	NA	NA	not addressed	NA	not addressed
Muenssinger et al.	2013a (2)	discrimination	tube/ bag	700 S: 560 D1 & D2: 70 each	∼17	NA	50–80	not addressed	NA	not addressed
Muenssinger et al.^a^	2013b	discrimination	tube/ balloon	800 S: 700 D: 100	**4.93**	NA	100 to tones 5 & 6: 72.22 MMN (tone 5 vs. 6): **50**	MMN: 220 + /- 50	S: 10.25 + /- 3.41 D: 11.16 + /- 3.75 MMN: 19.57 + /- 9.55	n. s.
Porcaro et al.	2006	simple	tube	200–350	**10.44–18.26**	tone: 1566 mHB: 6068	tone: 66.67 mHB: 66.67	tone: 206 + /- 21 mHB: 258 + /- 48	tone: 83 + /- 34 mHB: 175 + /- 75	not addressed
Preissl et al.	2001 (1)	simple	tube/ mask	6	2	NA	**63.63**	**219.33 + /- 32.44**	**16.03 + /- 10.21**	not addressed
Schleger et al.	2014	discrimination	tube/ bag	S: **∼654 tones/** **327 trials** D: **∼328 tones/** **82 trials**	12	NA	MMN: 73.91	early: 207 + /- 66 late: 427 + /- 64	NA	response latencies: −8.1 ms/ week (*t*(21) = −2.59, *p* = .017)
Schleger et al.	2018	simple	tube/ bag	**180** S: **135** D: **45**	6	NA	**75.00**	maternal family history of diabetes: 235 + /- 94 control: 269 + /- 73	not addressed	not addressed
Schleussner & Schneider	2004	simple	tube	500	**8.75**	NA	at least one component: 77.4 P1pm: 34.0 N1pm: 24.5 P2pm: 30.2 N2pm: 39.3 P3pm: 30.2	analysed range: 56–394	not addressed	P1pm, N1pm, P2pm, & N2pm: decrease of latencies with increasing GA (sig. in P2pm (*p* = 0.022) & N2pm (*p* = 0.026))
Schleussner et al.	2004	simple	tube	500	**8.75**	NA	NA	analysed range: 56–394	not addressed	negative correlation at 29–39 weeks GA
Schleussner et al.	2001	simple	tube	500	**8.75**	NA	56**.25**	29.86 weeks GA (*n* = 1): 294 30 weeks GA: 300 at term:157	NA	negative correlation at 27–39 weeks GA
Schneider et al.	2001	simple	tube	500	**8.75**	NA	**51.56**	analysed range: 56–294	< 25	not addressed
Sheridan et al.^b^	2010	discrimination	tube/ bag	long/ short 735/ 721 tone pairs (1470/ 1442 tones) S: 1250/ 1226 tones D: 220/ 216 tones	long: 14 short: 11	NA	75–80 long: S: 80, D: 75 short: S: 75, D: 80	long ISI S (early): **347.2 + /- 134.52** S (late): **550.4 + /- 143.50** D (early): **360.66 + /- 169.55** D (late): **684.8 + /- 63.36** short ISI S (early): **306.4 + /- 101.99** D (early): **366.8 + /- 147.24** D (late): **553.6 + /-0**	long ISI S (early): **-6.62 + /- 11.57** S (late): **-15.47 + /- 6.34** D (early): **-4.89 + /- 10.54** D (late): **-9.95 + /- 3.75** short ISI S (early): **4.06 + /- 10.03** D (early): **5.57 + /- 8.88** D (late): **7.8 + /- 0**	no
Zappasodi et al.	2001	discrimination	tube	230	**10.09**	200	50	200–300	NA	not addressed

**Note:** Items in bold have been calculated by the current authors. GA = gestational age, mean, NA = not available, S = standard, D = deviant, AM = amplitude modulated tones, mHB = maternal heartbeat. ^a^ Amplitude values were excluded from statistical analyses as only root mean squares were reported. ^b^ Amplitude values were excluded from statistical analyses as this was the only study with negative amplitude values. ^x^ Tone was inserted between trials for response recovery to visual stimuli; foetal auditory and foetal visual evoked responses are reported separately. ^z^ Discrimination in attempt to avoid habituation, but only responses to standards were discussed. * Criterion for foetal auditory evoked response.

## Data Availability

The data and R code that support the findings of the present review and meta-analysis are openly available in OSF at https://doi.org/10.17605/OSF.IO/CXRYF.

## Glossary

Functional magnetic resonance imaging (fMRI): Neuroimaging method which indirectly measures brain activity by assessing changes in cerebral blood flow which are based on the Blood Oxygen Level Dependency (BOLD) response.
Foetal magnetoencephalography (fMEG): Neuroimaging method which indirectly measures brain activity through changes in magnetic fields that are based on a weak current flow between neurons.
Foetal evoked response (fER): Changes in foetal brain activity in response to one/ more (external) stimuli.
