# Supplementary material for: The functional foetal brain: A systematic review and meta-analysis
Source: Dev Cogn Neurosci. 2026 Mar 17;79:101708. doi: 10.1016/j.dcn.2026.101708 (PMC13053791; doi:10.1016/j.dcn.2026.101708)
Supplement: Supplementary file 1 — Supplementary material [file mmc1.docx]

Supplementary material

# Results and discussion

## Descriptive statistics

### Data groups

Out of the 141 data groups, 133 investigated fER with fMEG and eight with fMRI. 97 data groups looked at foetal auditory perception while 44 data groups investigated foetal visual perception. Samples with maternal or foetal risk factors for atypical development were investigated in eight data groups. Two data groups included typically as well as atypically developing foetuses, and 131 data groups investigated only typically developing foetuses. 91 data groups followed a longitudinal study design with two or more sessions per participant during the last trimester of pregnancy while 50 data groups were cross-sectional studies with only one session per participant.

Taken together and in line with the results of Dunn et al. (2015), foetal auditory evoked responses (fAER) have been studied more often than foetal visual evoked responses (fVER). Further, foetuses with risk factors for atypical development have been investigated less than typically developing foetuses.

### Sample characteristics

When looking at all data groups, the sample size was on average *M* = 18.15 (*SD* = 16.04, range: 1 – 60) while only approximately 16 participants were finally analysed per data group (*M* = 15.89, *SD* = 13. 1, range: 0 – 56). As in Dunn et al. (2015), the present results are thus mainly based on studies with small sample sizes. This is particularly true for studies that were published earlier and for those using fMRI methodologies. The average attrition rate was 17.89 % (*SD* = 20.71, range: 0 – 72.5), which is, on average, lower than previously proposed for foetal fMRI and fMEG studies based on assumptions related to infant attrition (Reid & Dunn, 2021). It is also lower compared to other measures used to access cognitive abilities of foetuses and infants (Visibelli et al., 2024). This may be further reduced in subsequent studies if technological advances can make methodologies more robust against artefacts and if standardised protocols lead to a more successful way of eliciting fER (Dunn et al., 2015). Note that the attrition rate of data groups using fMRI methodologies were almost doubled compared to the attrition rate in fMEG data groups (*M* = 31.84, *SD* = 16.52 vs. *M* = 16.63, *SD* = 20.11; *t*(7.32) = 2.32, *p* = .052). This may be due to motion artefacts being more severe in fMRI compared to fMEG studies. The attrition rate was not explored in the previous review (Dunn et al., 2015). The present paper indicates that future studies in the field of fER should choose adequate sample sizes to account for the loss of participants during data acquisition and subsequent data processing.

The GA ranged from 25 to 41 weeks GA with a mean of *M* = 32.77 weeks (32 weeks and 5 days, *SD* = 13.16 days). All fMRI and most fMEG studies investigated fER only during the last trimester of pregnancy (≥ 28 weeks), but there were 13 fMEG studies that included foetuses from 27 weeks GA, one included foetuses from 26 weeks GA, and one from 25 weeks GA (see tables A2 & A4 in the appendix). The present review gives a general overview of at which GA fER have been investigated, independent of methodology whereas Dunn et al. (2015) focused on the descriptive comparison of the GA range between fMRI and fMEG studies.

### Stimulus characteristics

The average stimulus duration was 1795.01ms (*SD* = 2848.78, range: 33 – 10000) while the mean ISI was approximately 300 ms shorter and had a narrower range than the stimulus itself (*M* = 1492.18, *SD* = 788.87, range: 200 – 4,500). The ratio of the stimulus duration and ISI ranged from 1:24 to 1.5:1 (*M* ~ 1:3.4, *SD* = ~ 1:3.63), which is similar to the range reported in Dunn et al. (2015).

Several different stimulus types were used across the included studies (see figure 2).

visual

auditory

**Fig. 2.** The number of data groups per stimulus type. *Note*. NA = not available.

Among auditory fMEG studies, tone bursts were the most prominent stimuli which were defined as tones with a length of 100 ms or less (e. g. Lengle et al., 2001; Preissl et al., 2001). These were followed by pure tones with a length of 500 ms. Apart from Jardri et al. (2008), who used pure tones, fMRI studies used more complex auditory stimuli than fMEG studies, including Spanish guitar music (Moore et al., 2001), vibroacoustic stimulation (Fulford et al., 2004), the mother’s singing (Goldberg et al., 2020), or the maternal voice being played from a recording (Hykin et al., 1999; Jardri et al., 2012). The different complexity of auditory stimuli in fMRI compared to fMEG studies adds to the variation in sample sizes and GA ranges that Dunn et al. (2015) previously described as causing fundamental differences between the two methodologies.

Visual studies, on the other hand, showed less variability with light flashes being the most prominent choice followed by a light flash set. Light flashes are individual flashes (33/ 500 ms) with an ISI in between single flashes. A light flash set consists of a set of four light flashes (500 ms each) with an ISI in between individual flashes and an intertrial interval in between flash sets of four light flashes. Fulford et al. (2003), the only visual fMRI study, used a red LED cluster with a much higher stimulus duration (8000 ms). One study did not give any information about the stimuli for auditory or visual stimulation (Bisgin et al. 2019).

Visual studies used a wavelength of either 625 nm or 630 nm (*M* = 626.63, *SD* = 2.37) and an average light intensity of *M* = 7140.00 lux (*SD* = 2221.67, range: 4000 – 8800). Fulford et al. (2004) did not specify their wavelength apart from that it was within the red LED spectrum (620 - 750 nm).

Auditory studies used a frequency of 80 Hz to 2250 Hz (*M* = 689.21 Hz, *SD* = 198.49) and the stimuli were on average *M* = 107.75 dB loud (*SD* = 15.37, range: 78 - 120 dB). The frequency was on average *M* = 517.44 Hz for the standard stimulus (*SD* = 83.94, range: 500 – 1500) and *M* = 840.2 Hz for the (first) deviant stimulus (*SD* = 204.82, range: 500 – 2250). FMEG studies used a higher SPL than fMRI studies with a range of 85 to 120 and 85 to 100, respectively (*M* = 107.80 dB, *SD* = 12.39 vs. *M* = 94.58 dB, *SD* = 7.49; *t*(7.28) = -3.94, *p* = .005).

When compared to the studies summarised in Dunn et al. (2015), studies published after the previous review used similar values for the different stimulus characteristics, except for usually lower SPL in more recent studies. This is in line with the consensus of the field that higher SPL can be harmful to the foetus and are no longer recommended (Graven et al., 2000). More recent studies have also been more consistent with the stimulus frequency, typically using 500 Hz for the standard and 750 Hz for the first deviant stimulus. Apart from the frequency level, stimulus characteristics display wide variability between studies, making direct comparisons difficult.

### Paradigm characteristics

All fMRI studies used a block design in which stimulation blocks were alternated with blocks of silence. FMEG studies used either a simple approach with one stimulus being alternated with silence or a discrimination/ habituation paradigm with a standard and at least one deviant stimulus that were interspersed with silence.

As shown in figure 3, all visual fMEG studies used a simple approach while Bisgin et al. (2019) did not specify which paradigm they used for their visual and auditory sub studies.

**Fig. 3.** The number of data groups per study paradigm, separated for auditory and visual studies. *Note.* Simple = only one stimulus was presented, discrimination/ habituation = more than one stimulus was presented, block design = stimulation vs. silence, NA = not available.

Discrimination was the most common paradigm in auditory fMEG studies (65 data groups) with a standard and mainly one deviant tone. Draganova et al. (2018) and Muenssinger et al. (2013a), however, used both more than one deviant stimulus and Moser et al. (2021) divided their paradigm into two phases with a local deviant in the learning phase and a global deviant in the later test phase. Furthermore, Matuz et al. (2012) used a tone for response recovery from a visual stimulus that was presented four times in a row before the tone was played. Matuz et al. (2012) was also the only study that stimulated both modalities in one paradigm. Although habituation was the most prominent paradigm, most studies did not report response rate, response latency and/ or response amplitude values for the deviant stimuli (but see Draganova et al., 2018; Hartkopf et al., 2016; Muenssinger et al., 2013b; Sheridan et al., 2010).

There was also a large variability across included studies how stimuli were delivered to the foetus (see figure 4).

**Fig. 4.** The number of data groups per delivery method. *Note*. NA = not available.

While Bisgin et al. (2019) did not report their delivery method, all other auditory fMEG studies used an approach where the speaker was placed in a separate room and the sound was delivered to the participant through a tube and either a bag, a balloon, a mask, or a funnel on the maternal abdomen. Auditory fMRI studies, in contrast, used a variety of options which ranged from headphones on the maternal abdomen (Jardri et al., 2008, 2012; Moore et al. 2001) over a speaker with a pipe (Hykin et al., 1999), an acoustic stimulator (Fulford et al., 2004) to the mother singing during the scan (Goldberg et al., 2020).

Visual studies mostly used a fiber-optic cable with or without a woven panel on the participant’s end (e. g. Eswaran et al., 2004; McCubbin et al., 2007). Morin et al. (2015) used fiber-optic wires and a light pad while Fulford et al. (2003) chose a light tube for their fMRI study.

The included studies presented their stimuli on average *M* = 263 times (*SD* = 208.51), including the standard and any deviant stimuli that were used in discrimination paradigms. There was a wide range between studies which was influenced not only by the method (fMRI vs. fMEG), but also the modality (auditory vs. visual). While there was only a relatively small difference between number of auditory (*M* = 285.90, *SD* = 214.49) and visual presentations (*M* = 213.48, *SD* = 82.56; *t*(136.61) = 2.9, *p* = .004), fMRI studies presented their stimuli far less often than fMEG studies (*M* = 22.25, *SD* = 11.77 vs. *M* = 281.67, *SD* = 204.94; *t*(110.17) = -12.77, *p* < .001).

On average, the standard stimulus was presented *M* = 312.5 times (*SD* = 320.48, range: 38 - 1080) while the (first) deviant stimulus was presented only *M* = 59.79 times (*SD* = 48.41, range: 24 - 221). Deviant stimuli, which were only used in auditory studies, were played approximately 23.98 % (*SD* = 11.09) of the time in habituation paradigms with one deviant (range: 12 – 50) and in 33.33 % (*SD* = 0) of the trials in studies with more than one deviant. Note that only auditory fMEG studies used deviants whereas the calculations for the standard stimulus also include data from fMRI and visual fMEG studies.

The mean experimental time, including all stimulus presentations and ISI, was on average *M* = 9.43 min long (*SD* = 4.93, range: 2 – 26) with only slight variations between fMRI (*M* = 11.27, *SD* = 4.60) and fMEG (*M* = 8.19, *SD* = 3.06; *t*(7.5) = 1.86, *p* = .102), auditory (*M* = 8.91, *SD* = 3.08) and visual studies (*M* = 7.33, *SD* = 1.99; *t*(122.43) = 3.64, *p* < .001).

In sum, while fMRI and visual fMEG studies show little to no change in paradigm characteristics over time, more recent auditory fMEG studies are using more complicated study paradigms (but see Schleger et al., 2018). They are also more likely to present the stimulus more often and have longer experimental sessions than earlier studies.

### Study Quality

The quality ratings of the included studies, which are based on the average scores calculated from the quality assessment forms (see appendix A in supplementary material), are summarised in figure 5. Four of the included studies were rated as being “good” (8.7 %), 13 as “sufficient” (28.26 %), 27 as “insufficient” (58.7 %), and two as “poor” (4.35 %).

**Fig. 5.** The study quality for fMRI, visual and auditory fMEG studies. *Note.* The study quality was rated from 1 = very good to 6 = very poor. None of the included studies were either very good or very poor.

When analysing the data groups, the pattern is slightly different. 11 % as of the data groups were rated as being “good”, 50 % as “sufficient”, 35 % as “insufficient”, and 4 % as “poor”. This difference is due to how many data groups were extracted from individual studies which was based on if separate data was reported for separate GA groups and/ or experimental conditions. The mean quality rating of the data groups was *M* = 3.67 (*SD* = .45) which sits within the “sufficient” rating. Note that the quality assessment ranged from “very good” to “very poor”, while the overall ratings ranged only from “good” to “poor” (numeric range: 2.73 – 4.55).

The most common issues across studies were an incomplete description of the sample, missing information about methodologies and results, as well as (very) low sample sizes (e. g. *N* = 3; Jardri et al., 2008, 2012), which was particularly true for fMRI and earlier fMEG studies (e. g. Eswaran et al., 2002b; Fulford et al., 2003; Zappasodi et al., 2001). As earlier studies were largely feasibility studies, this is expected. In contrast, the results of the eleven more recent fMEG studies were based on an increased number of participants and technological advances such as improved algorithms, thus likely producing more reliable results. Providing quality ratings for studies of fAER and fVER potentially offers a new approach to understand differences between study results, even when similar sample, stimulus, and paradigm characteristics are present.

### Analysed trials

Matuz et al. (2012), Moore et al. (2001), and Sheridan et al. (2008) reported how many trials were included per participant (5-19, ≥ 70, ≥ 30, respectively), while all other studies provided no information or only the total number of analysed trials (*M* = 168, *SD* = 75.50, range: 30 - 237). There were also seven studies that reported the number of analysed standard trials which ranged from 46 to 1566 trials (*M* = 223.53, *SD* = 376.51). In total, only ten out of the 46 included studies gave any information about how many trials were included in their analyses (see tables A1, A3, & A5 in the appendix), showing no improvement since Dunn et al. (2015).

## Publication and selection bias

### Cumulative forest plots

Figures 6 and 7 show the cumulative forest plots for data groups that reported standard latency and standard amplitude values, respectively. Please note that both cumulative forest plots are not separated by GA. Instead, both list all data groups with different GA and experimental conditions in regard to the study they were extracted from.

**Estimate [95 %]**

243.20 [233.50, 252.90] 231.43 [208.04, 254.82] 240.58 [217.62, 263.55] 249.86 [225.40, 274.31] 251.82 [232.30, 271.33] 245.23 [224.63, 265.83] 260.81 [225.79, 295.83] 259.28 [228.81, 289.74] 271.11 [235.62, 306.59] 263.62 [228.65, 298.59] 270.57 [236.13, 305.02] 278.69 [243.46, 313.93] 277.21 [244.65, 309.76] 277.97 [247.76, 308.18] 276.19 [247.83, 304.55] 271.24 [242.99, 299.48] 268.90 [241.93, 295.87] 267.95 [242.43, 293.46] 262.37 [235.89, 288.84] 266.94 [240.28, 293.60] 270.61 [244.25, 296.97] 269.89 [244.72, 295.07] 267.20 [242.57, 291.84] 262.12 [236.54, 287.70] 265.14 [239.90, 290.37] 264.17 [239.84, 288.49] 261.08 [236.92, 285.24] 260.56 [237.24, 283.87] 260.69 [238.19, 283.20] 262.20 [240.24, 284.16] 260.64 [239.19, 282.09] 260.26 [239.47, 281.06] 256.57 [235.17, 277.98] 259.23 [237.83, 280.63] 258.52 [237.70, 279.35] 257.71 [237.41, 278.02] 261.27 [240.35, 282.20] 261.88 [241.48, 282.27] 261.20 [241.30, 281.11] 259.05 [239.20, 278.89] 257.74 [238.22, 277.26] 257.75 [238.69, 276.80] 256.96 [238.29, 275.63] 256.20 [237.91, 274.50] 258.98 [240.29, 277.67] 258.33 [240.00, 276.65] 261.20 [242.42, 279.99] 260.42 [241.97, 278.88] 262.73 [244.10, 281.36] 261.69 [243.33, 280.05] 263.87 [245.37, 282.36] 265.57 [247.12, 284.01] 267.50 [249.02, 285.99] 269.35 [250.85, 287.85] 270.82 [252.43, 289.20] 273.36 [254.64, 292.08] 273.71 [255.30, 292.11] 274.95 [256.70, 293.21] 275.48 [257.51, 293.45] 277.51 [259.39, 295.63] 279.46 [261.23, 297.68] 281.67 [263.22, 300.11] 283.53 [265.01, 302.05] 284.43 [266.11, 302.74] 286.01 [267.71, 304.31] 286.48 [268.43, 304.53] 286.94 [269.13, 304.74] 287.08 [269.53, 304.63] 286.03 [268.62, 303.44] 285.27 [268.05, 302.49] 284.30 [267.21, 301.38] 282.91 [265.85, 299.97] 282.04 [265.13, 298.96] 281.75 [265.05, 298.44] 281.77 [265.30, 298.24] 283.28 [266.76, 299.80] 284.37 [267.92, 300.81] 284.59 [268.35, 300.83] 282.89 [266.51, 299.26] 281.65 [265.30, 298.00] 280.27 [263.91, 296.64] 278.96 [262.59, 295.33] 278.43 [262.23, 294.64] 278.32 [262.31, 294.33] 278.25 [262.43, 294.08] 277.95 [262.30, 293.60] 278.12 [262.65, 293.60] 278.26 [262.96, 293.56] 277.29 [262.04, 292.54] 276.56 [261.42, 291.71] 275.38 [260.22, 290.54]


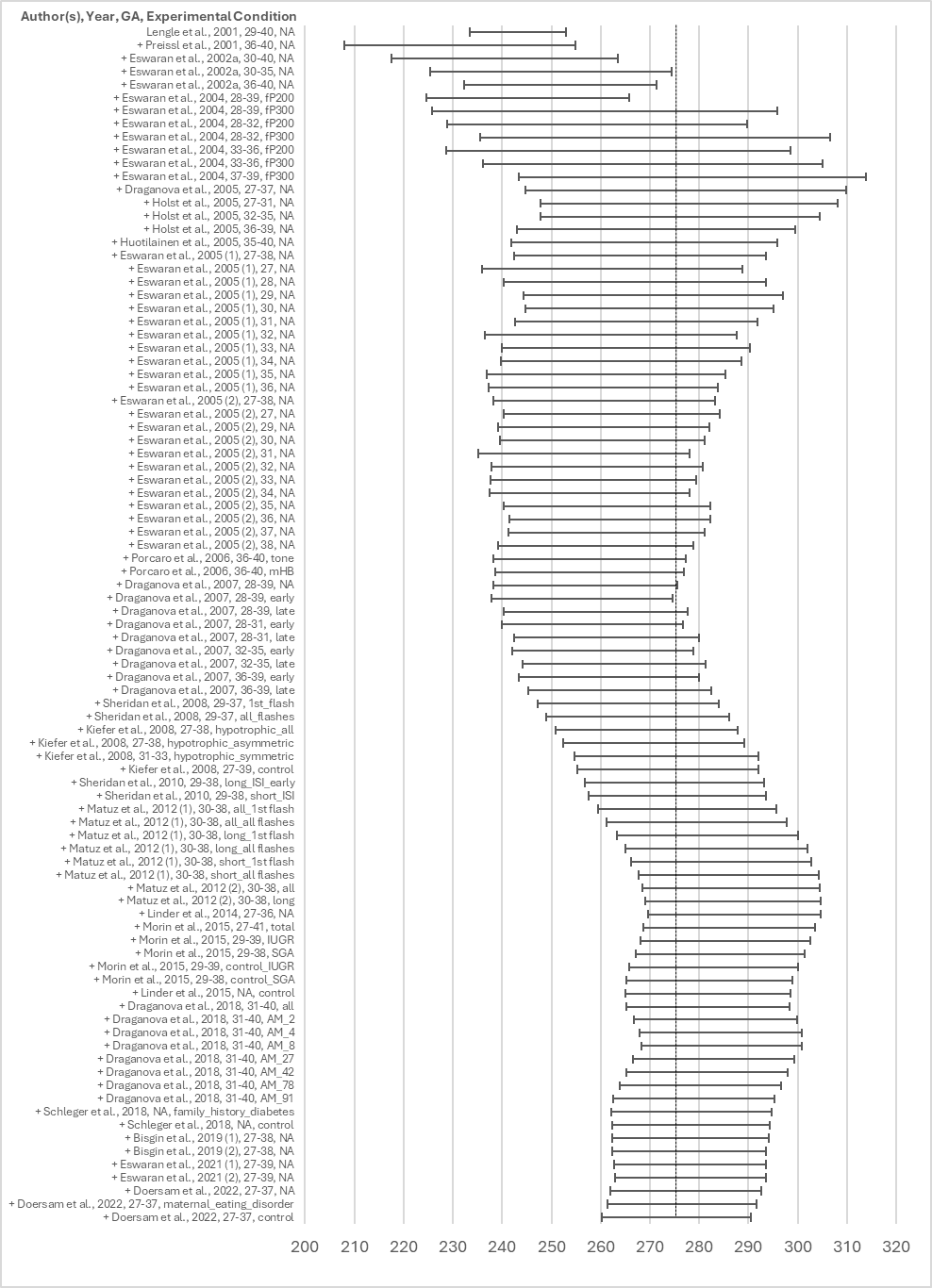


sturkop

200 210 220 230 240 250 260 270 280 290 300 310 320

**response latency (in ms)**

**Fig. 6.** Cumulative forest plot for standard latency. *Note.* GA = gestational age. “NA” in the left column refers to missing information or is used as a place holder if the separation into different experimental conditions was not applicable for a data group. The dashed line represents the weighted average standard latency of 275.38 ms which is slightly different to the descriptive mean of 273.97 ms. This is because each individual mean is weighted based on the sample size while they contributed equally to the descriptive mean.

**Estimate [95 %]**

11.89 [8.23, 15.55]

13.15 [9.42, 16.88]

13.23 [10.62, 15.84]

13.27 [10.98, 15.56]

13.59 [11.50, 15.68]

13.76 [11.73, 15.79]

13.40 [11.46, 15.34]

13.72 [11.84, 15.59]

14.28 [12.34, 16.23]

14.59 [12.76, 16.42]

15.43 [13.28, 17.58]

14.82 [12.61, 17.04]

14.36 [12.38, 16.34]

14.13 [12.30, 15.96]

14.92 [12.79, 17.04]

14.84 [12.89, 16.78]

15.18 [13.20, 17.17]

15.83 [13.67, 17.99]

16.25 [14.07, 18.42]

16.29 [14.27, 18.31]

17.69 [14.80, 20.58]

17.76 [14.99, 20.53]

18.80 [15.57, 22.03]

19.14 [15.94, 22.34]

19.51 [16.34, 22.68]

19.37 [16.31, 22.43]

19.61 [16.60, 22.61]

19.87 [16.93, 22.82]

19.71 [16.84, 22.57]

20.29 [17.33, 23.25]

20.49 [17.61, 23.38]

22.41 [17.85, 26.96]

22.66 [18.22, 27.09]

23.00 [18.65, 27.35]

23.08 [18.86, 27.31]

24.74 [19.51, 29.97]

0 10 20 30 40

**response amplitude (in fT)**

**Fig. 7.** Cumulative forest plot for standard amplitude. *Note.* GA = gestational age. “NA” in the left column refers to missing information or is used as a place holder if the separation into different experimental conditions was not applicable for a data group. The dashed line represents the average standard amplitude of 24.74 fT.

While the mean standard latency changed only about 30 ms over time, from 243.20 ms to 275.38 ms, the standard amplitude approximately doubled from 11.89 fT in 2001 to 24.74 fT in 2006. Afterwards, amplitude values were reported by only a few studies and those could not be included in the analyses (see paragraph 2.5.1). Furthermore, while the confidence intervals for the standard latency decreased from 2001 to 2022, their size changed only slightly for the standard amplitude. Figure 7 displays a gradual shift of the amplitude towards a higher average while the plot of the standard latency resembles a steadier pattern where the cumulative mean moved around the final average. The gradual increase in the mean amplitude could be due to attributes of the samples or changes in stimulus and paradigm characteristics which are further discussed in paragraphs 1.1.2 - 1.1.4. Enhanced methodologies in more recent studies, such as noise reduction systems, could have also contributed to the observed changes. These improvements could have also led to the smaller confidence intervals for the standard latency in more recent data groups.

### Egger’s regression test

Egger’s regression test for funnel plot asymmetry (Egger et al., 1997) was significant for the standard latency (*z* = 4.15, *p* < .001) and the LDN of the standard latency (*z* = -2.72, *p* = .007), indicating a possible publication bias for both variables. Neither the tests for the standard amplitude, nor for the MMN of the standard latency were significant (both *p* ≥ .187).

### Trim and fill method

The trim and fill method (Duval & Tweedie, 2000) did not find missing studies for the standard latency (*SE* = 5.56) but it found a substantial amount of heterogeneity between studies (tau^2^ = 5376.83, *SD* = 811.17; see figure 8). The average latency of *M* = 275.38 ms (*SE* = 7.73) was within a 95 % confidence interval of CI = 260.22 to 290.54 ms for the 91 data groups that could be included in the analysis. 19 data groups were deleted in the trim and fill plot (see figure 8).

**Fig. 8.** Trim and fill plot standard latency. *Note.* Each dot represents one data group. Black dots belong to data groups that were unaffected by the trim and fill method whereas white dots were deleted using this method. No additional data groups were created by the trim and fill method. The grey dashed lines represent the average response latency of 275.38 ms which is bounded by the standard error.

The trim and fill method identified twelve missing studies on the right side of the plot for the standard amplitude (*SE* = 3.80) which were inserted into the plot (see figure 9). The heterogeneity was tau^2^ = 282.82 (*SD* = 59.84) with a 95 % confidence interval of CI = 25.63 to 35.27 fT around a mean of *M* = 30.45 fT (*SE* = 2.46 fT) for the 48 analysed data groups.

**Fig. 9.** Trim and fill plot standard amplitude. *Note.* Each dot represents one data group. Black dots belong to data groups that were unaffected by the trim and fill method whereas grey circled dots were created by the trim and fill method to adjust for possible publication and selection biases. None of the data groups were deleted by the trim and fill method. The grey dashed lines represent the average response amplitude of 30.45 fT which is bounded by the standard error.

Dunn et al. (2015) suggested that the field of fER might be affected by publication and selection biases. However, they did not assess any measures of bias in their study. In the present study, Egger’s regression test suggests the presence of a publication bias for the standard latency, while figure 8 shows a relatively symmetrical distribution of the individual latencies around the mean. These findings suggest a rather weak publication bias for standard latencies. This result could, however, also be due to the different methodologies implemented by the different studies, e. g. various stimulus duration.

Figure 9 indicates a bias for the data groups that reported standard amplitude values with possibly missing values above the upper confidence interval (> 35.27 fT). Note, however, that only approximately half the number of data groups was included in the trim and fill test for the standard amplitude compared to the one for the standard latency (48 vs. 91) and that amplitude values from four studies had been excluded from the analyses (see paragraph 2.5.1 in the manuscript). Furthermore, latency values had been reported from 2001 to 2022 while amplitude values were only available until 2006. The trim and fill test also showed a much higher variability between data groups for the standard latency than for the standard amplitude. It is consequently difficult to determine if standard amplitude values were influenced by publication or selection biases, or if those values were affected by methodological and technical limitations that were present at the time.

## Multiple regression analyses

### Typical samples

As can be seen in tables S1 and S2, the standard latency in samples with typical development was mainly moderated by stimulus characteristics while the values for the standard amplitude were dependent on the stimulus type, the response rate, and the GA. Given that the study identification number and the GA groups were part of the best fitting model for the standard response amplitude with many significant factor levels, there was a substantial amount of variability for the amplitude across studies that went beyond the sample, stimulus, and paradigm characteristics used as possible predictors. This could, however, also have been due to the relatively low number of data groups that reported amplitude values in addition to the large amount of missing information in the dataset in general.

**Table S1**

Multiple regression analyses – results for standard response latency.

|  | typical samples | auditory studies | visual studies |
| --- | --- | --- | --- |
| model with continuous variables | - ISI (*t* = -2.04, *p* = .043)* - light intensity (*t* = -3.49, *p* < .001)*** - study quality (*t* = 2.33, *p* = .021)* - all others *p* ≥ 2.878 | - all *p* ≥ .115 | - number of total presentations (*t* = 2.24, *p* = .032)* - mean experimental time (*t* = -2.08, *p* = .045)* - all others *p* ≥ .117 |
| model with categorical variables | - stimulus type: 2 Hz & 4 Hz AM tones vs. tone bursts (both   *t* = 2.15, *p* = .037)*   - all others *p* ≥ .160 | - stimulus type: 2 Hz & 4 Hz AM tones vs. tone bursts (*t* = 2.58, *p* = .016 & *t* = 2.59, *p* = .016)* - GA groups: 31 vs. 27 WG (*t* = -2.23, *p* = .035)*, 37-40 vs. 27 WG (*t* = -2.35, *p* = .027)* - all others *p* ≥ .093 | - GA groups: 28 vs. 27 WG   (*t* = 2.45, *p* = .031)*, 29 vs. 27 WG (*t* = 2.33, *p* = .038)*, & 33 vs. 27 WG (*t* = 2.24, *p* = .045)*   - all others *p* ≥ .075 |
| model with all variables | - all *p* ≥ .152 | - all *p* ≥ .127 | - all *p* ≥ .104 |
| model with best fit (all variables) | - response rate (*t* = 1.47,   *p* = .145)   - stimulus type: flash set   (*t* = 5.74, *p* < .001)*; 4 Hz & 27 Hz AM tones (*t* = 2.02, *p* = .046 & *t* = -2.43, *p* = .017)*   - adjusted *R*^2^ = .239 | - stimulus duration (*t* = -1.19, *p* = .243) - number of total presentations (*t* = 1.47, *p* = .151) - mean experimental time (*t* = -0.98, *p* = .335) - GA (.005 < *p* < .043)* - stimulus type: 2 Hz AM tones (*t* = 2.86, *p* = .007)*; 4 Hz AM tones (*t* = 2.93, *p* = .006)* - paradigm (*p* ≥ .217) - adjusted *R*^2^ = .259 | - GA groups (.002 < *p* < .045)* - adjusted *R*^2^ = .433 |

**Note:** Asterix * and *** indicate significant effects for *p* < .05 and *p* < .001, respectively. ISI = interstimulus interval, AM tones = amplitude modulated tones, GA = gestational age, WG = weeks of gestation.

**Table S2**

Multiple regression analyses – results for standard response amplitude.

|  | typical samples | auditory studies | visual studies |
| --- | --- | --- | --- |
| model with continuous variables | - ISI (*t* = 2.7, *p* = .008)** - standard frequency (*t* = 2.46, *p* = .015)* - response rate (*t* = 2.17, *p* = .032)* - all others *p* ≥ .107 | - ISI (*t* = 2.33, *p* = .022)* - standard frequency (*t* = 2.31, *p* = .023)* - response rate (*t* = 2.00, *p* = .049)* - all others *p* ≥ .192 | - ratio stimulus duration/ ISI   (*t* = -3.20, *p* = .003)**   - all others *p* ≥ .068 |
| model with categorical variables | - stimulus type: pure tones vs. tone bursts (*t* = 8.11, *p* < .001)*** - study identification number   (<.001 < *p* < .044)*   - GA groups (<.001 < *p* < .05)* - all others *p* ≥ .07 | - stimulus type: pure tones vs. tone bursts (*t* = 52.17, *p* < .001)*** - study identification number   (*p* < .001)***   - GA groups (*p* < .001)*** - all others *p* ≥ .124 | - study identification number (<.001 < *p* < .03)* - GA groups (<.001 < *p* < .03)* - all others *p* ≥ .058 |
| model with all variables | - standard frequency (*t* = 8.56, *p* < .001)*** - response rate (*t* = 2.73, *p* = .01)** - GA groups (<.001 < *p* < .047)* - all others *p* ≥ .061 | - standard frequency (*t* = 136.33, *p* < .001)*** - mean GA (*t* = 11.61, *p* < .001)*** - study quality (*t* = -2.37, *p* = .031)* - study identification number   (.011 < *p* < .041)*   - GA groups (<.001 < *p* < .009)** - all others *p* ≥ .06 | - ISI (*t* = -4.08, *p* = .002)** - ratio stimulus duration/ ISI   (*t* = -4.89, *p* < .001)***   - mean experimental time   (*t* = 2.80, *p* = .017)*   - study identification number: Eswaran et al., 2005   (*t* = -2.27, *p* = .044)*   - GA groups (<.001 < *p* < .023)* - all others *p* ≥ .050 |
| model with best fit (all variables) | - response rate (*t* = 2.96, *p* = .005)** - standard latency (*t* = 1.54, *p* = .132) - study identification number   (<.001 < *p* < .029)*   - GA group (<.001 < *p* < .045)* - stimulus type: pure tones vs. tone bursts   (*t* = 9.34, *p* < .001)***   - adjusted *R*^2^ = .723 | - mean GA (*t* = 14.97, *p* < .001)*** - study identification number   (*p* <.001)***   - GA groups (<.001 < *p* = .001)*** - stimulus type: pure tones vs. tone bursts: *t* = 167.71, *p* < .001)*** - adjusted *R*^2^ = .999 | - GA groups (<.001 < *p* < .049)* - adjusted *R*^2^ = .891 |

**Note:** Asterix *, **, and *** indicate significant effects for *p* < .05, *p <* .01, and *p* < .001, respectively. ISI = interstimulus interval, GA = gestational age.

### Auditory and visual studies

When analysing auditory and visual data groups separately, there were significant differences between the results, which can be seen in tables S1 and S2.

One of the main differences was the influence of stimulus characteristics on fAER and fVER. The stimulus characteristics moderated latency and amplitude values only in the models for auditory data groups, not in any of the visual models. This might have been caused by the relatively large variability of auditory stimuli while visual stimuli were more alike. Since the variance of stimulus characteristics in visual studies was low, the covariance between stimulus characteristics and fVER had to be low as well because without sufficient variability of the individual variables, they could not share a substantial covariance with each other (Eledum, 2017). Since a correlation is a normalised covariance (e. g. Asuero et al., 2006), a low covariance leads to a low correlation coefficient. This statistical relationship between variance and correlation could explain the non-significant result for stimulus characteristics in visual models in the present dataset.

Both fAER and fVER were significantly moderated by the GA which was also a significant predictor for all four models with the best fit. While the two auditory models with the best fit also included several other stimulus and paradigm characteristics, the GA alone explained 43.27 % of the variance in latency values and 89.14 % of amplitude variance of fVER (see tables S1 and S2). This finding underlines the importance of reporting sufficient information about the GA of the participants, including the mean and standard deviation of separately analysed groups to understand differences in the results between studies based on different GA ranges.

Another common significant predictor for both fAER and fVER was the study identification number. It was not included in the models with the best fit, but it showed several significant factor levels in the linear models for both auditory and visual data groups. Hence, there were significant differences between amplitude values of different studies that went beyond the analysed stimulus and paradigm characteristics. The large amount of missing information might have also contributed to this finding. Further studies are needed to see which (additional) variables influence amplitude values of fAER and fVER.

The amplitude values of fER to standard stimuli were also influenced by the ISI in at least one of the analysed models. Visual stimuli showed further moderation by the ratio of stimulus duration to ISI and the mean experimental time. This heightens not only the importance of choosing an appropriate ISI, but also the relevance of reporting the ISI and the stimulus duration in order that meaningful comparisons of amplitude values between studies may take place.

## Moderating analyses

No moderating analyses were performed for areas with changed brain activity following auditory or visual stimulation. This was due to too much heterogeneity between the seven included fMRI studies. Because of the amount of missing data, moderating analyses could also not be performed for the response rate.

Data groups with atypical development could not be separately analysed because of too much missing information, but all other three subsamples (typical samples, auditory studies, visual studies) were independently analysed. The results are summarised in table S3.

**Table S3**

Results from moderating analyses.

|  | typical samples | auditory studies | visual studies |
| --- | --- | --- | --- |
| latency | - light intensity (*z* = -4.00, *p* = .001)*** - study quality (*z* = 2.54, *p* = .011)* - wavelength (*z* = 4.22, *p* < .001)*** - GA groups: 29-37 vs. 27 WG (*z* = 2.09, *p* = .037)*, 30-38 vs. 27 WG (*z* = 2.52, *p* = .012)* - stimulus type: flash set > tone bursts (*z* = 5.68, *p* < .001)***;   flash set > pure tones; flash set > light flashes; 2 - 8 Hz AM tones > all others; 27 - 91 Hz < all others   - paradigm: bimodal > simple & habituation - delivery method: fiber-optic cable/ woven panel > all others - ISI (*z* = -1.95, *p* = .052) - all others: *p* ≥ .063 | - SPL (*z* = 1.99, *p* = .047)* - GA groups - stimulus type: flash set > tone bursts, pure tones, & light flashes; 2 - 8 Hz AM tones > all others; 27 - 91 Hz < all others - paradigm: habituation > simple - delivery method (fiber-optic cable/ woven panel vs. all others - all others: *p* ≥ .093 | - light intensity (*z* = -4.24, *p* < .001)*** - number of total presentations   (*z* = 3.65, *p* = < .001)***   - study design: longitudinal vs. cross-sectional (*z* = 2.37, *p* = .018)* - stimulus type: light flashes vs. flash set (*z* = -4.90, *p* < .001)*** - delivery method: fiber-optic cable/ woven panel > fiber-optic cable > fiber-optic wires/ light pad - stimulus durations (*z* = 2.13, *p* .033)* - ratio stimulus duration/ ISI (*z* = 1.73, *p* = .084) - all others: *p* ≥ .105 |
| amplitude | - publication year (*z* = 2.96, *p* = .003)** - ISI (*z* = 4.07, *p* < .001)*** - mean experimental time (*z* = 2.51,   *p* = .012)*   - study identification number: Porcaro et al. (2006) > all others (*z* = 4.08,   *p* < . 001)***   - stimulus type: pure tones vs. tone bursts & light flashes (*z* ≥ 4.45,   *p* < .001)***   - GA group: 35 WG > all others - delivery methods: tube > all others - stimulus durations (*z* = 1.67, *p* .095) - all others: *p* ≥ .129 | - publication date (*z* = 2.42, *p* = .015)* - ISI (*z* = 3.08, *p* = .002)** - study identification number: Porcaro et al. (2006) > all others (*z* = 4.08,   *p* < . 001)***   - stimulus type: pure tones vs. tone bursts (*z* = 3.52, *p* < .001)*** - all others: *p* ≥ .115 | - publication date (*z* = 2.62, *p* = .009)** - number of total presentations   (*z* = -2.62, *p* = .009)**   - study quality (*z* = 2.62, *p* = .009)** - stimulus duration (*z* = -2.62,   *p* = .009)**   - ISI (*z* = 2.62, *p* = .009)** - ratio stimulus duration/ ISI (*z* = -2.62, *p* = .009)** - all others: *p* ≥ .919 (continuous variables) |

**Note:** Asterix *, **, and *** indicate significant effects for *p* < .05, *p <* .01, and *p* < .001, respectively. GA = gestational age, AM tones = amplitude modulated tones, ISI = interstimulus interval, WG = weeks of gestation, SPL = sound pressure level. The significant difference between studies using light of 625 nm and 630 nm was unlikely to be caused by the wavelength as a difference of 5 nm was not visible for the human eye. Only Draganova et al. (2018) used amplitude modulated tones and those were used in a habituation paradigm which could have led to the discrepancies compared to other auditory stimuli that were also used in simple designs.

Both the response latency and the response amplitude show more significant moderating effects for visual compared to auditory data groups. This suggests that the methodology impacts the results of visual studies to a greater extent than it does in studies using auditory stimuli. This can be explained by how the external stimulus reaches the foetal sensory organs, eyes and ears. Auditory stimuli may be less impacted by foetal movements and the thickness of maternal tissues when contrasted to visual stimuli. In contrast, the appearance of visual stimuli is affected by maternal tissue thicknesses and is also highly susceptible to changes in foetal positioning (Leov, 2024). If the foetus was oriented away from the light source, the stimulus would not be seen while there would be little to no change in the perception of auditory stimuli.

Apart from the GA groups, all categorical variables had only one factor level in the amplitude analyses which made it impossible to analyse moderating effects of categorical variables on amplitude values. Furthermore, all included data groups of the amplitude analyses for visual studies were drawn from two studies which constrains the generalisation of these results. Further studies are required to fully understand how amplitude values are moderated by sample, stimulus, and paradigm characteristics.

## Second order moderating analyses

Modality (auditory vs. visual) and the mean GA were separately added as second order moderators to the beforementioned analyses. The results of these analyses are summarised in table S4.

**Table S4**

Results from second order moderating analyses.

| modality (auditory vs. visual) | GA (latency only) |
| --- | --- |
| latency (all samples):   - mean GA (z = -1.78, *p* = .075)   latency (typical samples):   - ratio stimulus duration/ ISI (*z* = 2.22, *p* = .026)* - number of total presentations (*z* = 2.07, *p* = .039)* - ISI (*p* = .052) - publication year (*p* = .062) - stimulus duration (*p* = .072)   amplitude (all samples & typical samples):   - study quality (*z* = -2.49, *p* = .013)* - publication year (*z* = -2.23, *p* = .026)* - number of total presentations (*z* = -2.02, *p* = .043)* - ISI (*p* = .064) - mean experimental time (*p* = .085) | all samples:   - delivery method (*z* = 2.24, *p* = .025)* - standard frequency (*z* = 1.99, *p* = .046)* - SPL (*p* = .055) - response rate (*p* = .070)   typical samples:   - number of total presentations (*z* = -2.47, *p* = .013)* - delivery method (*p* = .066)   auditory studies:   - standard frequency (*z* = 1.99, *p* = .046)* - delivery method (*p* = .055) - SPL (*p* = .055) - response rate (*p* = .070) |

**Note:** Asterix * indicate significant effects for *p* < .05. All amplitude results show smaller amplitude values for visual compared to auditory stimuli. Only six to 13 data groups were included in the subsample analyses for the GA on latency values.

The small number of data groups could have caused the non-significant findings while the moderations might have been significant if more studies had reported the mean GA and had measured the response amplitude to the standard stimulus. Whether the mean GA had significant effects on response amplitude could not be explored as there was only one data group that reported both the mean GA and the standard response amplitude.

Taken together, both the modality as well as the mean GA seem to affect the moderating effects of several of the investigated variables for the standard latency and the standard amplitude. The modality and the mean GA should therefore be considered as higher order moderators in future studies.

# References

References marked with an asterisk indicate studies included in the current review and meta-analysis.

Asuero, A. G., Sayago, A., & González, A. G. (2006). The correlation coefficient: An overview. *Critical Reviews in Analytical Chemistry*, *36*(1), 41-59. https://doi.org/10.1080/10408340500526766

*Bisgin, N., Wilson, J. D., Murphy, P., Siegel, E. R., Lowery, C. L., & Eswaran, H. (2019). Relationship between fetal behavioral states and auditory and visual stimulation. *2019 IEEE EMBS International Conference on Biomedical & Health Informatics (BHI)*, 1-4. IEEE. https://doi.org/10.1109/BHI.2019.8834519

*Doersam, A. F., Moser, J., Throm, J., Weiss, M., Zipfel, S., Micali, N., Preissl, H., & Giel, K. E. (2022). Maternal eating disorder severity is associated with increased latency of foetal auditory event‐related brain responses. *European Eating Disorders Review*, *30*(1), 75-81. https://doi.org/10.1002/erv.2870

*Draganova, R., Eswaran, H., Murphy, P., Huotilainen, M., Lowery, C., & Preissl, H. (2005). Sound frequency change detection in fetuses and newborns, a magnetoencephalographic study. *Neuroimage*, *28*(2), 354-361. https://doi.org/10.1016/j.neuroimage.2005.06.011

*Draganova, R., Eswaran, H., Murphy, P., Lowery, C., & Preissl, H. (2007). Serial magnetoencephalographic study of fetal and newborn auditory discriminative evoked responses. *Early Human Development*, *83*(3), 199-207. https://doi.org/10.1016/j.earlhumdev.2006.05.018

*Draganova, R., Schollbach, A., Schleger, F., Braendle, J., Brucker, S., Abele, H., Kagan, K. O., Wallwiener, D., Fritsche, A., Eswaran, H., & Preissl, H. (2018). Fetal auditory evoked responses to onset of amplitude modulated sounds. A fetal magnetoencephalography (fMEG) study. *Hearing Research*, *363*, 70-77. https://doi.org/10.1016/j.heares.2018.03.005

Dunn, K., Reissland, N., & Reid, V. M. (2015). The functional foetal brain: A systematic preview of methodological factors in reporting foetal visual and auditory capacity. *Developmental Cognitive Neuroscience, 13*, 43-52. https://doi.org/10.1016/j.dcn.2015.04.002

Duval, S., & Tweedie, R. (2000). A nonparametric “trim and fill” method of accounting for publication bias in meta-analysis. *Journal of the American Statistical Association, 95*(449), 89-98. https://doi.org/10.1080/01621459.2000.10473905

Egger, M., Smith, G. D., Schneider, M., & Minder, C. (1997). Bias in meta-analysis detected by a simple, graphical test. *British Medical Journal, 315*(7109), 629-634. https://doi.org/10.1136/bmj.315.7109.629

Eledum, H. Y. (2017). A Monte Carlo Study of the Effects of Variability and Outliers on the Linear Correlation Coefficient. *Journal of Modern Applied Statistical Methods, 16*(2), 231-255. https://doi.org/10.22237/jmasm/1509495180

*Eswaran, H., Lau, C., Murphy, P., Siegel, E. R., Preissl, H., & Lowery, C. (2021). Tracking evoked responses to auditory and visual stimuli in fetuses exposed to maternal high‐risk conditions. *Developmental Psychobiology*, *63*(1), 5-15. https://doi.org/10.1002/dev.22008

*Eswaran, H., Lowery, C. L., Wilson, J. D., Murphy, P., & Preissl, H. (2004). Functional development of the visual system in human fetus using magnetoencephalography. *Experimental Neurology*, *190*(Suppl. 1), 52-58. https://doi.org/10.1016/j.expneurol.2004.04.007

*Eswaran, H., Lowery, C. L., Wilson, J. D., Murphy, P., & Preissl, H. (2005). Fetal magnetoencephalography—a multimodal approach. *Developmental Brain Research*, *154*(1), 57-62. https://doi.org/10.1016/j.devbrainres.2004.10.003

*Eswaran, H., Preissl, H., Wilson, J. D., Murphy, P., Robinson, S. E., Rose, D. F., Vrba, J., & Lowery, C. L. (2002a). Short-term serial magnetoencephalography recordings offetal auditory evoked responses. *Neuroscience Letters*, *331*(2), 128-132. https://doi.org/10.1016/S0304-3940(02)00859-5

*Eswaran, H., Wilson, J. D., Preissl, H., Robinson, S. E., Vrba, J., Murphy, P., Rose, D. F., & Lowery, C. L. (2002b). Magnetoencephalographic recordings of visual evoked brain activity in the human fetus. *The Lancet*, *360*(9335), 779-780. https://doi.org/10.1016/S0140-6736(02)09905-1

*Fulford, J., Vadeyar, S. H., Dodampahala, S. H., Moore, R. J., Young, P., Baker, P. N., James, D. K., & Gowland, P. A. (2003). Fetal brain activity in response to a visual stimulus. *Human Brain Mapping*, *20*(4), 239-245. https://doi.org/10.1002/hbm.10139

*Fulford, J., Vadeyar, S. H., Dodampahala, S. H., Ong, S., Moore, R. J., Baker, P. N., James, D. K., & Gowland, P. (2004). Fetal brain activity and hemodynamic response to a vibroacoustic stimulus. *Human Brain Mapping*, *22*(2), 116-121. https://doi.org/10.1002/hbm.20019

Graven, S. N. (2000). Sound and the developing infant in the NICU: conclusions and recommendations for care. *Journal of Perinatology*, *20*(1), S88-S93.

*Goldberg, E., McKenzie, C. A., de Vrijer, B., Eagleson, R., & de Ribaupierre, S. (2020). Fetal response to a maternal internal auditory stimulus. *Journal of Magnetic Resonance Imaging*, *52*(1), 139-145. https://doi.org/10.1002/jmri.27033

*Hartkopf, J., Schleger, F., Weiss, M., Hertrich, I., Kiefer-Schmidt, I., Preissl, H., & Muenssinger, J. (2016). Neuromagnetic signatures of syllable processing in fetuses and infants provide no evidence for habituation. *Early Human Development*, *100*, 61-66. https://doi.org/10.1016/j.earlhumdev.2016.04.002

*Holst, M., Eswaran, H., Lowery, C., Murphy, P., Norton, J., & Preissl, H. (2005). Development of auditory evoked fields in human fetuses and newborns: a longitudinal MEG study. *Clinical Neurophysiology*, *116*(8), 1949-1955. https://doi.org/10.1016/j.clinph.2005.04.008

*Huotilainen, M., Kujala, A., Hotakainen, M., Parkkonen, L., Taulu, S., Simola, J., Nenonen, J., Karjalainen, M., & Näätänen, R. (2005). Short-term memory functions of the human fetus recorded with magnetoencephalography. *Neuroreport*, *16*(1), 81-84. https://doi.org/10.1097/00001756-200501190-00019

*Hykin, J., Moore, R., Duncan, K., Clare, S., Baker, P., Johnson, I., Bowtell, R., Mansfield, P., & Gowland, P. (1999). Fetal brain activity demonstrated by functional magnetic resonance imaging. *The Lancet*, *354*(9179), 645-646. https://doi.org/10.1016/S0140-6736(99)02901-3

*Jardri, R., Houfflin-Debarge, V., Delion, P., Pruvo, J. P., Thomas, P., & Pins, D. (2012). Assessing fetal response to maternal speech using a noninvasive functional brain imaging technique. *International Journal of Developmental Neuroscience*, *30*(2), 159-161. https://doi.org/10.1016/j.ijdevneu.2011.11.002

*Jardri, R., Pins, D., Houfflin-Debarge, V., Chaffiotte, C., Rocourt, N., Pruvo, J. P., Steinling, M., Delion, P., & Thomas, P. (2008). Fetal cortical activation to sound at 33 weeks of gestation: a functional MRI study. *Neuroimage*, *42*(1), 10-18. https://doi.org/10.1016/j.neuroimage.2008.04.247

*Kiefer, I., Siegel, E., Preissl, H., Ware, M., Schauf, B., Lowery, C., & Eswaran, H. (2008). Delayed maturation of auditory-evoked responses in growth-restricted fetuses revealed by magnetoencephalographic recordings. *American Journal of Obstetrics and Gynecology*, *199*(5), 503-e1. https://doi.org/10.1016/j.ajog.2008.04.014

*Lengle, J. M., Chen, M., & Wakai, R. T. (2001). Improved neuromagnetic detection of fetal and neonatal auditory evoked responses. *Clinical Neurophysiology*, *112*(5), 785-792. https://doi.org/10.1016/S1388-2457(01)00532-6

Leov, J. N. (2024). *A light in the dark; an investigation of fetal visual perception* [Doctoral dissertation, The University of Waikato]. The University of Waikato Research Commons. https://hdl.handle.net/10289/17038

*Linder, K., Schleger, F., Ketterer, C., Fritsche, L., Kiefer-Schmidt, I., Hennige, A., Häring, H. U., Preissl, H., & Fritsche, A. (2014). Maternal insulin sensitivity is associated with oral glucose-induced changes in fetal brain activity. *Diabetologia*, *57*, 1192-1198. https://doi.org/10.1007/s00125-014-3217-9

*Linder, K., Schleger, F., Kiefer-Schmidt, I., Fritsche, L., Kümmel, S., Heni, M., Weiss, M., Häring, H. U., Preissl, H., & Fritsche, A. (2015). Gestational diabetes impairs human fetal postprandial brain activity. *The Journal of Clinical Endocrinology & Metabolism*, *100*(11), 4029-4036. https://doi.org/10.1210/jc.2015-2692

*Matuz, T., Govindan, R. B., Preissl, H., Siegel, E. R., Muenssinger, J., Murphy, P., Ware, M., Lowery, C. L., & Eswaran, H. (2012). Habituation of visual evoked responses in neonates and fetuses: a MEG study. *Developmental Cognitive Neuroscience*, *2*(3), 303-316. https://doi.org/10.1016/j.dcn.2012.03.001

*McCubbin, J., Murphy, P., Eswaran, H., Preissl, H., Yee, T., Robinson, S. E., & Vrba, J. (2007). Validation of the flash-evoked response from fetal MEG. *Physics in Medicine & Biology*, *52*(19), 5803. https://doi.org/10.1088/0031-9155/52/19/005

*Moore, R. J., Vadeyar, S., Fulford, J., Tyler, D. J., Gribben, C., Baker, P. N., James, D., & Gowland, P. A. (2001). Antenatal determination of fetal brain activity in response to an acoustic stimulus using functional magnetic resonance imaging. *Human Brain Mapping*, *12*(2), 94-99. https://doi.org/10.1002/1097-0193(200102)12:2<94::AID-HBM1006>3.0.CO;2-E

*Morin, E. C., Schleger, F., Preissl, H., Braendle, J., Eswaran, H., Abele, H., Brucker, S., & Kiefer‐Schmidt, I. (2015). Functional brain development in growth‐restricted and constitutionally small fetuses: a fetal magnetoencephalography case–control study. *BJOG: An International Journal of Obstetrics & Gynaecology*, *122*(9), 1184-1190. https://doi.org/10.1111/1471-0528.13347

*Moser, J., Schleger, F., Weiss, M., Sippel, K., Semeia, L., & Preissl, H. (2021). Magnetoencephalographic signatures of conscious processing before birth. *Developmental Cognitive Neuroscience*, *49n*, 100964. https://doi.org/10.1016/j.dcn.2021.100964

*Muenssinger, J., Matuz, T., Schleger, F., Draganova, R., Weiss, M., Kiefer-Schmidt, I., Wacker-Gussmann, A., Govindan, R. B., Lowery, C. L., Eswaran, H., & Preissl, H. (2013a). Sensitivity to auditory spectral width in the fetus and infant–an fMEG study. *Frontiers in Human Neuroscience*, *7*, 917. https://doi.org/10.3389/fnhum.2013.00917

*Muenssinger, J., Matuz, T., Schleger, F., Kiefer‐Schmidt, I., Goelz, R., Wacker‐Gussmann, A., Birbaumer, N., & Preissl, H. (2013b). Auditory habituation in the fetus and neonate: an fMEG study. *Developmental Science*, *16*(2), 287-295. https://doi.org/10.1111/desc.12025

*Porcaro, C., Zappasodi, F., Barbati, G., Salustri, C., Pizzella, V., Rossini, P. M., & Tecchio, F. (2006). Fetal auditory responses to external sounds and mother's heart beat: Detection improved by Independent Component Analysis. *Brain Research*, *1101*(1), 51-58. https://doi.org/10.1016/j.brainres.2006.04.134

*Preissl, H., Eswaran, H., Murphy, P., Wilson, J. D., Robinson, S. E., Vrba, J., Fife, A. A., Tilotson, M., & Lowery, C. L. (2001). Recording of temporal-spatial biomagnetic signals over the whole maternal abdomen with SARA-auditory fetal brain responses. *Biomedical Engineering / Biomedizinische Technik, 46*(2), 191-193. https://doi.org/10.1515/bmte.2001.46.s2.191

Reid, V. M., & Dunn, K. (2021). The fetal origins of human psychological development. *Current Directions in Psychological Science*, *30*(2), 144-150. https://doi.org/10.1177/09637214209844

*Schleger, F., Landerl, K., Muenssinger, J., Draganova, R., Reinl, M., Kiefer-Schmidt, I., Weiss, M., Wacker‐Gussmann, A., Huotilainen, M., & Preissl, H. (2014). Magnetoencephalographic signatures of numerosity discrimination in fetuses and neonates. *Developmental Neuropsychology*, *39*(4), 316-329. https://doi.org/10.1080/87565641.2014.914212

*Schleger, F., Linder, K., Walter, L., Heni, M., Brändle, J., Brucker, S., Pauluschke-Fröhlich, J., Weiss, M., Häring, H. U., Preissl, H., & Fritsche, A. (2018). Family history of diabetes is associated with delayed fetal postprandial brain activity. *Frontiers in Endocrinology*, *9*, 673. https://doi.org/10.3389/fendo.2018.00673

*Schleussner, E., & Schneider, U. (2004). Developmental changes of auditory-evoked fields in fetuses. *Experimental Neurology*, *190*(Suppl. 1), 59-64. https://doi.org/10.1016/j.expneurol.2004.04.008

*Schleussner, E., Schneider, U., Arnscheidt, C., Kähler, C., Haueisen, J., & Seewald, H. J. (2004). Prenatal evidence of left–right asymmetries in auditory evoked responses using fetal magnetoencephalography. *Early Human Development*, *78*(2), 133-136. https://doi.org/10.1016/j.earlhumdev.2004.03.005

*Schleussner, E., Schneider, U., Kausch, S., Kähler, C., Haueisen, J., & Seewald, H. J. (2001). Fetal magnetoencephalography: a non-invasive method for the assessment of fetal neuronal maturation. *British Journal of Obstetrics and Gynaecology*, *108*(12), 1291-1294. https://doi.org/10.1016/S0306-5456(01)00292-3

*Schneider, U., Schleussner, E., Haueisen, J., Nowak, H., & Seewald, H. J. (2001). Signal analysis of auditory evoked cortical fields in fetal magnetoencephalography. *Brain Topography*, *14*(1), 69-80. https://doi.org/10.1023/A:1012519923583

*Sheridan, C., Draganova, R., Ware, M., Murphy, P., Govindan, R., Siegel, E. R., Eswaran, H., & Preissl, H. (2010). Early development of brain responses to rapidly presented auditory stimulation: a magnetoencephalographic study. *Brain and Development*, *32*(8), 642-657. https://doi.org/10.1016/j.braindev.2009.10.002

*Sheridan, C. J., Preissl, H., Siegel, E. R., Murphy, P., Ware, M., Lowery, C. L., & Eswaran, H. (2008). Neonatal and fetal response decrement of evoked responses: a MEG study. *Clinical Neurophysiology*, *119*(4), 796-804. https://doi.org/10.1016/j.clinph.2007.11.174

Specialist Unit for Review Evidence. Questions to Assist with the Critical Appraisal of Cross-Sectional Studies. Retrieved December 18, 2023, from SURE-CA-form-for-Cross-sectional_2018.pdf

Visibelli, E., Porru, A., Lucangeli, D., Butterworth, B., & Benavides-Varela, S. (2024). Neural indicators of numerical abilities in the infant human brain: A systematic review. *Developmental Review*, *74*, 101150. https://doi.org/10.1016/j.dr.2024.101150

*Zappasodi, F., Tecchio, F., Pizzella, V., Cassetta, E., Romano, G. V., Filligoi, G., & Rossini, P. M. (2001). Detection of fetal auditory evoked responses by means of magnetoencephalography. *Brain Research*, *917*(2), 167-173. https://doi.org/10.1016/S0006-8993(01)02901-8

# Appendix

## Appendix A – quality assessment form

| Study: | |
| --- | --- |
| 1. Does the study address a clearly focused question?   Consider: Population, outcomes. |  |
| 1. Were participants fairly selected?   Consider: Eligibility criteria, sources, & selection of participants. |  |
| 1. Is the sample well described?   Consider: Maternal and foetal characteristics, such as maternal age, GA, pregnancy complications, etc. | *N*:  maternal age:  mother healthy/ medical conditions:  GA:  normal/ complications:  ethnicity:  foetal sex:  overall: |
| 1. Is the study design clearly stated? – stimulus characteristics | stimulus type:  stimulus duration:  ISI:  ratio stimulus duration/ ISI: calculated  frequency/ wavelength:  intensity:  overall: |
| 1. Is the study design clearly stated? – paradigm characteristics | paradigm:  delivery method:  number of presentations:  total time:  overall: |
| 1. Is the methodology well described?   Consider: Technical specifications, such as sampling rate, filter. | scanner/ scanner & channels (fMEG):  sampling rate:  trial length (fMEG):  offline filter (fMEG):  pre-processing steps:  overall: |
| 1. Has the sample size been estimated a priori? |  |
| 1. Analysed sample size | *N* = |
| 1. Are all relevant outcome measures given? | number of analysed trials:  response rate:  latency (early & late):  amplitude:  activated area (fMRI):  age effect:  overall: |
| 1. Are the results well described?   Consider: Mean & *SD*, range |  |
| 1. Does the study provide raw data/ averages for every participant? |  |
| 1. Is any sponsorship/ conflict of interest reported? | sponsorship:  conflict:  overall: |
| Summary: | |

**Note:** The quality assessment form was based on the Specialist Unit for Review Evidence (SURE) checklist for cross-sectional studies (2018). GA = gestational age, *N* = sample size, ISI = interstimulus interval, fMEG = foetal magnetoencephalography, fMRI = functional magnetic resonance imaging, *SD* = standard deviation.

## Appendix B – quality assessment – fMRI studies

| authors | date | focused question | participants selection | description of sample | stimulus  charac-  teristics | paradigm charac-teristics | description of metho-  dology | sample size estimation | analysed sample size | outcome measures given | description of results | raw data/ averages available | sponsorship/ conflict of interest | overall quality rating |
| --- | --- | --- | --- | --- | --- | --- | --- | --- | --- | --- | --- | --- | --- | --- |
| Fulford et al. | 2004 | 4 | 6 | 4 | 2 | 1 | 1 | NA | 4 | 3 | 2 | 2 | NA | 4 |
| Goldberg et al. | 2020 | 2 | NA | 1 | 3 | 1 | 1 | NA | 5 | 3 | 2 | 3 | 4 | 3 |
| Hykin et al. | 1999 | 4 | NA | 3 | 2 | 1 | 1 | NA | 6 | 3 | 3 | 3 | 4 | 4 |
| Jardri et al. | 2012 | 4 | 2 | 4 | 1 | 1 | 2 | NA | 6 | 3 | 3 | NA | NA | 4 |
| Jardri et al. | 2008 | 2 | 2 | 4 | 1 | 1 | 1 | NA | 6 | 4 | 3 | 3 | 4 | 4 |
| Moore et al. | 2001 | 2 | 3 | 3 | 2 | 1 | 1 | NA | 5 | 3 | 3 | 2 | 4 | 3 |
| Fulford et al. | 2003 | 3 | 6 | 4 | 2 | 1 | 1 | NA | 5 | 3 | 4 | 3 | NA | 4 |

**Note:** Quality rating: 1 = very good, 2 = good, 3 = sufficient, 4 = insufficient, 5 = poor, 6 = very poor, NA = not available (not stated/ missing). The rating regarding if the study addresses a clearly focused question (focused question) was not considered for the average quality rating of the study (overall quality rating) as all research questions were rated in relation to the topic of the present review while the investigation of foetal evoked responses was often not the objective of the included studies. Instead, especially earlier studies looked at feasibility of new designs or analysis techniques. The rating for the focused question was only included for the sake of completeness.

| authors | date | focused question | participants selection | description of sample | stimulus charac-  teristics | paradigm charac-teristics | description of metho-  dology | sample size estimation | analysed sample size | outcome measures given | description of results | raw data/ averages available | sponsorship/ conflict of interest | overall quality rating |
| --- | --- | --- | --- | --- | --- | --- | --- | --- | --- | --- | --- | --- | --- | --- |
| Bisgin et al. | 2019 (1) | 3 | NA | 3 | NA | NA | 1 | NA | 3 | 3 | 2 | NA | NA | 5 |
| Eswaran et al. | 2021 (1) | 1 | 2 | 2 | 2 | 1 | 1 | NA | 1 | 2 | 1 | NA | 4 | 2 |
| Eswaran et al. | 2004 | 4 | NA | 5 | 1 | 1 | 1 | NA | 5 | 2 | 1 | 2 | 4 | 3 |
| Eswaran et al. | 2005 (1) | 2 | NA | 4 | 1 | 1 | 1 | NA | 5 | 2 | 1 | 2 | 4 | 3 |
| Eswaran et al. | 2002b | 2 | NA | 5 | 1 | 1 | 2 | NA | 5 | 4 | 5 | NA | 1 | 4 |
| Matuz et al. | 2012 (1) | 1 | NA | 1 | 1 | 1 | 2 | NA | 3 | 1 | 1 | 2 | 1 | 2 |
| McCubbin et al. | 2007 | 5 | NA | 4 | 1 | 1 | 1 | NA | 3 | 4 | 5 | NA | 4 | 4 |
| Morin et al. | 2015 | 4 | 2 | 4 | 1 | 1 | 1 | NA | 3 | 4 | 1 | 2 | 1 | 2 |
| Sheridan et al. | 2008 | 2 | NA | 3 | 1 | 1 | 2 | NA | 3 | 4 | 3 | 2 | 4 | 3 |

## Appendix C – quality assessment – visual fMEG studies

**Note:** Quality rating: 1 = very good, 2 = good, 3 = sufficient, 4 = insufficient, 5 = poor, 6 = very poor, NA = not available (not stated/ missing). The rating regarding if the study addresses a clearly focused question (focused question) was not considered for the average quality rating of the study (overall quality rating) as all research questions were rated in relation to the topic of the present review while the investigation of foetal evoked responses was often not the objective of the included studies. Instead, especially earlier studies looked at feasibility of new designs or analysis techniques. The rating for the focused question was only included for the sake of completeness.

## Appendix D – quality assessment – auditory fMEG studies

| authors | date | focused question | participants selection | description of sample | stimulus charac-  teristics | paradigm charac-teristics | description of metho-  dology | sample size estimation | analysed sample size | outcome measures given | description of results | raw data/ averages available | sponsorship/ conflict of interest | overall quality rating |
| --- | --- | --- | --- | --- | --- | --- | --- | --- | --- | --- | --- | --- | --- | --- |
| Bisgin et al. | 2019 (2) | 3 | NA | 3 | 6 | NA | 1 | NA | 3 | 3 | 2 | NA | NA | 5 |
| Doersam et al. | 2022 | 3 | 6 | 2 | 1 | 1 | 5 | NA | 5 | 5 | 2 | NA | 1 | 4 |
| Draganova et al. | 2005 | 3 | NA | 5 | 1 | 2 | 1 | NA | 5 | 3 | 1 | 2 | 4 | 4 |
| Draganova et al. | 2007 | 2 | NA | 5 | 1 | 1 | 1 | NA | 4 | 1 | 1 | NA | 4 | 4 |
| Draganova et al. | 2018 | 3 | NA | 5 | 1 | 2 | 1 | NA | 3 | 2 | 1 | NA | 1 | 3 |
| Eswaran et al. | 2021 (2) | 1 | 2 | 2 | 1 | 2 | 1 | NA | 1 | 2 | 1 | NA | 4 | 2 |
| Eswaran et al. | 2005 (2) | 3 | NA | 4 | 1 | 1 | 1 | NA | 5 | 3 | 1 | 2 | 4 | 3 |
| Eswaran et al. | 2002a | 2 | NA | 4 | 1 | 1 | 1 | NA | 5 | 3 | 5 | 2 | 4 | 4 |
| Hartkopf et al. | 2016 | 4 | 4 | 2 | 1 | 1 | 1 | NA | 3 | 4 | 2 | NA | NA | 4 |
| Holst et al. | 2005 | 1 | NA | 3 | 1 | 1 | 1 | NA | 4 | 2 | 1 | NA | 4 | 3 |
| Huotilainen et al. | 2004 | 2 | 5 | 3 | 1 | 4 | 1 | NA | 4 | 2 | 2 | 2 | NA | 4 |
| Kiefer et al. | 2008 | 1 | 3 | 3 | 1 | 1 | 2 | NA | 5 | 2 | 1 | NA | NA | 4 |
| Lengle et al. | 2001 | 2 | NA | 4 | 1 | 1 | 3 | NA | 5 | 2 | 3 | 2 | 4 | 4 |
| Linder et al. | 2014 | 3 | NA | 2 | 1 | 1 | 1 | NA | 5 | 5 | 3 | NA | 1 | 4 |
| Linder et al. | 2015 | 3 | NA | 2 | 1 | 1 | 3 | NA | 5 | 5 | 5 | NA | 1 | 4 |
| Matuz et al. | 2012 (2) | 1 | NA | 1 | 1 | 2 | 1 | NA | 5 | 4 | 5 | 4 | 1 | 4 |
| Moser et al. | 2021 | 2 | 4 | 2 | 1 | 1 | 2 | NA | 2 | 4 | 4 | 4 | 1 | 3 |
| authors | date | focused question | participants selection | description of sample | stimulus charac-  teristics | paradigm charac-teristics | description of metho-  dology | sample size estimation | analysed sample size | outcome measures given | description of results | raw data/ averages available | sponsorship/ conflict of interest | overall quality rating |
| Muenssinger et al. | 2013a (1) | 4 | NA | 3 | 1 | 1 | 1 | NA | 5 | 6 | 6 | NA | 1 | 4 |
| Muenssinger et al. | 2013a (2) | 3 | NA | 3 | 2 | 1 | 1 | NA | 5 | 6 | 6 | NA | 1 | 4 |
| Muenssinger et al. | 2013b | 1 | NA | 3 | 1 | 1 | 1 | NA | 3 | 2 | 1 | NA | 4 | 3 |
| Porcaro et al. | 2006 | 3 | 5 | 3 | 1 | 2 | 1 | NA | 5 | 2 | 1 | NA | 4 | 3 |
| Preissl et al. | 2001 | NA | NA | 5 | 1 | 1 | 1 | NA | 4 | 3 | 4 | 2 | 4 | 4 |
| Schleger et al. | 2014 | 5 | NA | 3 | 1 | 1 | 1 | NA | 4 | 3 | 3 | NA | 4 | 4 |
| Schleger et al. | 2018 | 3 | 2 | 1 | 1 | 1 | 3 | NA | 4 | 4 | 2 | NA | 1 | 3 |
| Schleussner & Schneider | 2004 | 3 | NA | 4 | 1 | 1 | 2 | NA | 5 | 4 | 4 | NA | 4 | 4 |
| Schleussner et al. | 2004 | 1 | NA | 5 | 1 | 1 | 2 | NA | 3 | 4 | 5 | NA | 4 | 4 |
| Schleussner et al. | 2001 | 2 | 6 | 3 | 1 | 1 | 2 | NA | 3 | 5 | 5 | NA | NA | 4 |
| Schneider et al. | 2001 | 4 | NA | 3 | 1 | 1 | 1 | NA | 4 | 4 | 5 | NA | NA | 4 |
| Sheridan et al. | 2010 | 4 | NA | 3 | 1 | 1 | 2 | NA | 4 | 2 | 2 | 2 | 4 | 3 |
| Zappasodi et al. | 2001 | 5 | 2 | 2 | 1 | 1 | 1 | NA | 5 | 4 | 5 | NA | NA | 4 |

**Note:** Quality rating: 1 = very good, 2 = good, 3 = sufficient, 4 = insufficient, 5 = poor, 6 = very poor, NA = not available (not stated/ missing). The rating regarding if the study addresses a clearly focused question (focused question) was not considered for the average quality rating of the study (overall quality rating) as all research questions were rated in relation to the topic of the present review while the investigation of foetal evoked responses was often not the objective of the included studies. Instead, especially earlier studies looked at feasibility of new designs or analysis techniques. The rating for the focused question was only included for the sake of completeness.

## Appendix E – used R packages

The packages “base”, “readxl”, “tidyverse”, “metafor”, and “esc” were used for descriptive analyses while the “meta” package was used for meta-analyses. In addition to those, the packages “car”, “forcats”, “MASS”, and “leaps” were used for multiple regression analyses.
